# Chronic respiratory disease surveys in adults in low- and middle-income countries: A systematic scoping review of methodological approaches and outcomes

**DOI:** 10.7189/jogh.11.04026

**Published:** 2021-06-19

**Authors:** Nik Sherina Hanafi, Dhiraj Agarwal, Soumya Chippagiri, Evelyn A Brakema, Hilary Pinnock, Aziz Sheikh, Su-May Liew, Chiu-Wan Ng, Rita Isaac, Karuthan Chinna, Li Ping Wong, Norita Hussein, Ahmad Ihsan Abu Bakar, Yong-Kek Pang, Sanjay Juvekar, Ee Ming Khoo

**Affiliations:** 1 Department of Primary Care Medicine, Faculty of Medicine, University of Malaya, Kuala Lumpur, Malaysia; 2 KEM Hospital Research Centre, Pune, India; 3 Christian Medical College, Vellore, India; 4 Department of Public Health and Primary Care, Leiden University Medical Centre, Leiden, the Netherlands; 5 NIHR Global Health Research Unit on Respiratory Health (RESPIRE), Usher Institute, The University of Edinburgh, Edinburgh, UK; 6 Department of Social and Preventive Medicine, Faculty of Medicine, University of Malaya, Kuala Lumpur, Malaysia; 7 School of Medicine, Faculty of Health and Medical Sciences, Taylor’s University, Subang Jaya, Malaysia; 8 Pusrawi Hospital Sdn Bhd, Kuala Lumpur, Malaysia; 9 Department of Medicine, Faculty of Medicine, University of Malaya, Kuala Lumpur, Malaysia

## Abstract

**Background:**

Chronic respiratory diseases (CRDs) contribute significantly towards the global burden of disease, but the true prevalence and burden of these conditions in adults is unknown in the majority of low- and middle-income countries (LMICs). We aimed to identify strategies – in particular the definitions, study designs, sampling frames, instruments, and outcomes – used to conduct prevalence surveys for CRDs in LMICs. The findings will inform a future RESPIRE Four Country ChrOnic Respiratory Disease (4CCORD) study, which will estimate CRD prevalence, including disease burden, in adults in LMICs.

**Methods:**

We conducted a scoping review to map prevalence surveys conducted in LMICs published between 1995 and 2018. We followed Arksey and O’Malley’s six-step framework. The search was conducted in OVID Medline, EMBASE, ISI Web of Science, Global Health, WHO Global Index Medicus and included three domains: CRDs, prevalence and LMICs. After an initial title sift, eight trained reviewers undertook duplicate study selection and data extraction. We charted: country and populations, random sampling strategies, CRD definitions/phenotypes, survey procedure (questionnaires, spirometry, tests), outcomes and assessment of individual, societal and health service burden of disease.

**Results:**

Of 36 872 citations, 281 articles were included: 132 from Asia (41 from China). Study designs were cross-sectional surveys (n = 260), cohort studies (n = 11) and secondary data analysis (n = 10). The number of respondents in these studies ranged from 50 to 512 891. Asthma was studied in 144 studies, chronic obstructive pulmonary disease (COPD) in 112. Most studies (100/144) based identification of asthma on symptom-based questionnaires. In contrast, COPD diagnosis was typically based on spirometry findings (94/112); 65 used fixed-ratio thresholds, 29 reported fixed-ratio and lower-limit-of-normal values. Only five articles used the term ‘phenotype’. Most studies used questionnaires derived from validated surveys, most commonly the European Community Respiratory Health Survey (n = 47). The burden/impact of CRD was reported in 33 articles (most commonly activity limitation).

**Conclusion:**

Surveys remain the most practical approach for estimating prevalence of CRD but there is a need to identify the most predictive questions for diagnosing asthma and to standardise diagnostic criteria.

Chronic respiratory diseases (CRDs) contribute significantly to the national, regional and global burden of disease [1]. Although the morbidity and mortality of such conditions are estimated to be high in low- and middle-income countries (LMICs), there is concern that there is little robust data on the true prevalence of asthma and chronic obstructive pulmonary disease (COPD) in these countries [2,3]. Accurate data on prevalence, including disease burden, are needed to inform health care policy on prioritising care and targeting risk factors particularly for conditions that are preventable and treatable in primary care.

CRD encompasses several conditions. Most common are asthma and COPD; but others such as bronchiectasis, post-tuberculosis, interstitial lung disease and lung cancer are potentially important causes of morbidity in LMICs. In poorly-resourced primary health care systems in LMICs the conditions often remain undiagnosed as limited access to health care is compounded by insufficient attention to the conditions and lack of diagnostic capabilities in the facilities [4,5]. Determining the prevalence of asthma and COPD in the community remains a challenge because of the poor sensitivity and specificity of widely used questionnaire-based research tools [3,6]. Objective testing with spirometry may be a challenge in community-based epidemiological surveys [7], particularly in LMICs.

Scoping reviews are used to map the literature available and to identify potential gaps in the evidence base [8]. We conducted a systematic scoping review on the prevalence of CRDs to address the following questions with respect to prevalence studies on CRDs:

1. What surveys on the prevalence of asthma, COPD and other CRDs have been undertaken in LMICs?
2. What definitions, questionnaires, tests, diagnostic processes and outcomes for CRDs did the surveys employ?
3. How was the socio-economic burden (from a societal or healthcare perspective) of asthma, COPD and other CRDs estimated in these surveys?
4. What strategies have been used to identify phenotypes of asthma and COPD, or to identify the causes of ‘other CRD’?

We intend to use the findings of this scoping review to inform the methodology of a proposed RESPIRE survey in 4 Countries estimating prevalence and burden of Chronic Respiratory Disease in adults in LMICs [4CCORD study].

## METHODS

We followed the six-step framework for undertaking scoping reviews described by Arksey and O’Malley [9] which has been widely employed [10,11]. The full protocol for this systematic scoping review has been published [12]. The research questions and methods are summarised below.

### Search strategy

We developed a comprehensive search strategy assisted by information librarians. The search strategy (MEDLINE search strategy detailed in Table S1 in the **Online Supplementary Document**) comprised of keywords and subject headings (eg, MeSH) that were used to identify studies within three domains – CRDs, prevalence (including ‘burden’ or ‘cost’ in the title/abstract) and LMICs. The strategy was ﬁrst conducted in OVID MEDLINE. We then tailored and applied the strategy in EMBASE, ISI WoS, Global Health and WHO Global Index Medicus. We included studies published between 1995, when the Global Initiative on Asthma (GINA) was launched [13], and 2018. We did not limit our search to any language.

### Study selection

We included articles reporting community-based prevalence studies of CRDs that were published in academic journals. Eight members of the research team were involved in the initial study selection. As a scoping review, our search criteria were sensitive rather than specific. We anticipated many hits, so we carried out the selection process in three stages:

- A single reviewer performed initial title sift to exclude obviously irrelevant titles. Two reviewers (DA, NSH) conducted this individually on separate parts of the dataset.
- Screening of titles and abstracts (DA and NSH worked on separate datasets)
- Full text screening of potentially relevant articles (DA, NSH, HP, SC, SS or EAB)

To ensure better understanding of the studies’ methodological framework, at each stage we undertook a training process of duplicate screening of 200 citations. We compared initial decisions, resolved disagreements, and refined the inclusion and exclusion criteria in discussion with three arbiters (HP, EMK and SJ). This was repeated until we were satisfied that the selection criteria were clear, and researchers were achieving at least 90% agreement. At all stages, articles were grouped according to their relevance (relevant, not relevant or unsure); researchers were instructed to use an ‘unsure’ category if there was any doubt about relevance and these articles were then discussed within the team and a decision made. The selection criteria and definitions applied are detailed in **Table 1** [12].

**Table 1:** Inclusion and exclusion criteria [12].

Criterion	Inclusion and exclusion criteria
Population	We included surveys on general populations of adults (typically ≥18 years but used different thresholds according to age of majority which may vary in different countries). Surveys that included both adults and children were included but those that focused entirely on children were excluded. We excluded surveys on populations with known CRDs or respiratory diseases symptoms (for example: attendees at a respiratory clinic).
Screening procedure	We included surveys that determined the prevalence of asthma, COPD or other CRD using questionnaires, clinical examination, spirometry and/or other tests. We also included the prevalence of chronic respiratory symptoms and phenotypes.
Disease definitions	We included surveys that used definitions of CRD from globally recognised guidelines: asthma [14], COPD [15] or other CRD [7]. We defined ‘chronic’ respiratory symptoms as symptoms (such as cough, wheezing and shortness of breath) that have persisted for more than three months, or recurred in ‘attacks’. We did not include surveys on acute respiratory conditions such as pneumonia or active TB, therapeutic interventions, pharmaco-economics/cost analyses of medication or specific treatments, quality of disease management, assessment of inhaler technique, comparison between drug regimens, and health economic analyses (though we included prevalence studies that included assessments of socio-economic burden (eg, CRD-related time off work).
Burden of disease	We included population-level surveys of symptom burden, use of health care resources or societal burden (eg, absenteeism, loss of earnings).
Phenotypes	We included surveys that detected phenotypes of asthma, COPD or the overlap between these conditions.
Setting	We focused our review on low- or middle-income countries (LMICs) classified by the Organisation for Economic Cooperation and Development at the time of the survey. We included surveys in high-income countries only if the survey was also conducted in LMICs, eg, the BOLD study [16].
Study design	We included population or community surveys that aimed to determine the prevalence of one or more CRDs. The survey procedures included questionnaires, clinical examination, lung function tests (spirometry) or other tests (skin prick tests). We excluded randomised controlled trials, case-control studies and systematic reviews.

### Data charting

In line with scoping review methodology, we did not assess the quality of the individual studies because we aimed to map the evidence and not to summarise the study results nor to analyse disease risks and prevalence rates. We piloted and refined a customised data extraction form. Six reviewers (DA, NSH, SC, SS, HP or EAB) independently extracted data into the extraction form. Any ambiguities were resolved within the group. Studies from the same study population were reported as independent studies, with reference to the main article if indicated. Our research questions focused on identifying the process (rather than the outcomes) of undertaking surveys of CRD in LMICs. We made one attempt to contact authors of included articles for additional information, copies of questionnaires or study procedures if they were not otherwise available.

### Summarising and reporting the results

We summarised the data focusing on: 1) the strategies used to identify randomly sampled populations in LMICs; 2) the disease deﬁnitions used (which may vary over time); 3) the questionnaires used and tests performed to detect asthma, COPD and/or other CRDs; 4) the individual, societal and health care burden of CRD, and the risk factors for disease and 5) surveys that addressed contemporary understanding of asthma/COPD phenotypes. We held an investigators’ meeting with RESPIRE partners (from Bangladesh, India, Malaysia, Pakistan and Edinburgh, UK), where we discussed the findings. We followed the Preferred Reporting Items for Systematic reviews and Meta-Analyses extension for. Scoping Reviews (PRISMA-ScR) [17].

## RESULTS

**Figure 1:**
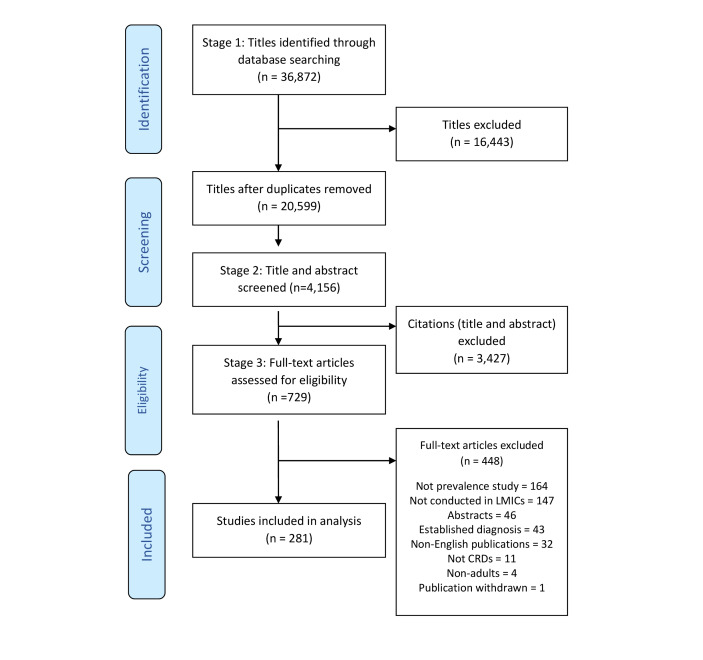
Preferred Reporting Items for Systematic Reviews And Meta-Analyses (PRISMA) flowchart for study selection process.

The literature search yielded 36 872 citations from five databases. After screening 729 full text articles, 281 were included in this scoping review (**Figure 1**). The study characteristics are detailed in Table S2 in the **Online Supplementary Document**.

### Question 1: What surveys on the prevalence of asthma, COPD and other CRDs have been undertaken in LMICs?

**Figure 2:**
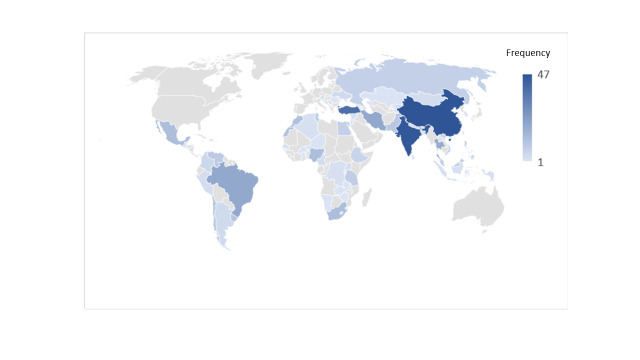
Distribution of chronic respiratory disease prevalence studies.

The 281 publications on CRD prevalence surveys in LMCs, published between 1995 and 2018, reported studies conducted in a total of 70 countries. Most publications were based on surveys conducted in Asia (n = 133 publications) with China, India and Turkey having 42, 35 and 30 publications, respectively (Table S1 in the **Online Supplementary Document**). This is followed by publications reported from the African (n = 45) and European continents (n = 39) (**Figure 2**). The number of publications reporting the prevalence of CRDs increased steadily over the years, from 11 between 1995-1999 to 98 between 2015-2018. Some studies, such as the BOLD study [16], was carried out in high-income countries as well as LMICs.

Of the 281 publications, 181 sampled from general populations, 57 recruited specific groups (clinic attendees or staff, university students and staff, elderly, women, men, athletes, smokers, non-smokers, aboriginal people and those with family history of asthma) and 43 were based on occupational groups. In this review, only surveys that screened for asthma, COPD and/or other CRDs were included. Surveys for specific occupational lung disease were excluded. Details are in Appendix S2 in the **Online Supplementary Document**.

The study designs of the 281 studies varied: cross-sectional surveys (n = 260), cohort surveys (n = 11) and secondary data analyses (n = 10). The cross-sectional surveys were typically house-to-house or community surveys (n = 178), at worksites (n = 40), surveys in clinics or health care facilities (n = 20), in universities (n = 8), telephone (n = 7) and postal surveys (n = 3); one postal survey was followed by a house-to-house survey due to initial low response rate.

The number of respondents in each study ranged from 50 to 512 891. Ten publications reported sample sizes of 100 000 or more; five in India [18-22], three in China [23-25] and two in multiple countries [26,27]. 101 studies had fewer than 1000 respondents. Response rates were reported in 128 surveys, out of which 91 achieved at least 80%.

### Question 2. What definitions, questionnaires, tests and diagnostic processes and outcomes for CRDs did the surveys employ?

**Figure 3:**
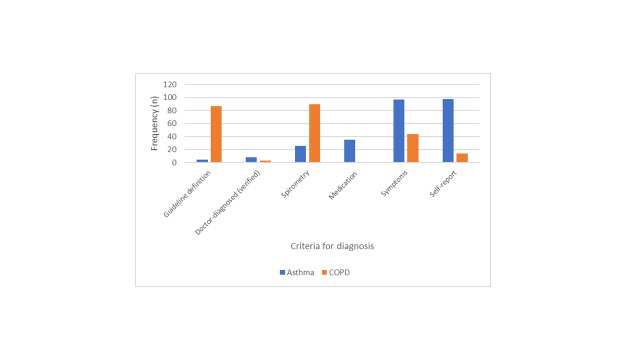
Criteria Used for Diagnosis of Asthma and COPD.

**Definition of diseases:** Surveys used various definitions for both asthma and COPD. **Figure 3** contrasts the approach for asthma and COPD.

For asthma diagnosis, most surveys used self-reported diagnosis (n = 98), or a questionnaire survey of symptoms (n = 97). Other methods were use of asthma medication (n = 35), spirometry (n = 26), verification through records (n = 8) and reference to GINA guidelines (n = 5). These methods were sometimes used in combination.

In marked contrast, for COPD diagnosis, 90 articles based the diagnosis on spirometry. Of these, 59 were based on a fixed Forced Expiratory Volume in one second/Forced Vital Capacity (FEV1/FVC) ratios, 28 used both fixed ratio and lower limits of normal (LLN) and three used only lower limit of normal as diagnostic criteria. Lower limit of normal was used in more recent studies (published since 2008). Other methods, which were sometimes used in combination, were according to GOLD guidelines (n = 87), symptoms (n = 44), self-reported diagnosis (n = 14) and doctor’s diagnosis, verified through records (n = 3).

**Questionnaires:** Most studies used validated questionnaires such as the European Community Respiratory Health Survey (ECRHS) (n = 58), American Thoracic Society Respiratory Questionnaire (ATS) (n = 43), International Union against Tuberculosis and Lung Disease (IUATLD) (n = 23) and the Medical Research Council Respiratory Questionnaire (MRC) (n = 14), either in totality, adapted or modified. The BOLD (n = 30) and PLATINO (n = 12) surveys used questionnaires derived from both the ECRHS and ATS questionnaires.

Common symptoms explored in the surveys were cough, wheezing, expectoration, dyspnea, breathlessness or phlegm as well nasal symptoms of itching, obstruction, sneezing, and secretion.

**Spirometry or peak flow meter:** Baseline spirometry was measured in 167 studies; of these, 109 also reported post-bronchodilator readings, usually to salbutamol (albuterol) in doses ranging from 100 to 400 µg. Two surveys used ipratropium and two used Combivent^TM^. Most studies reported on spirometry findings of obstructive patterns; only 22 studies reported prevalence of restriction. One study included chest x-ray findings to measure the prevalence of pneumoconiosis [28]. Two studies carried out in Malawi and Thailand [29,30] reported prevalence of restrictive lung disease using European normal values that could be an overestimation while four studies used modified values or lower limits of normal to suit local population [31-34].

Peak flow was measured in 13 surveys. Of these, five measured only peak flow without doing spirometry; four were worksite surveys [35-38] and one was a community survey exploring the effects of air quality [39].

Other diagnostic tests (each reported in one study) included exhaled CO level, DNA extraction, genetic biomarkers, pollution monitoring, RAST test, rhinomanometry, residue concentrations of pesticides in urine, serum assay for *C pneumoniae*, urine cotinine as well as water contact and fecal contaminants.

**Outcomes:** Most of the studies (n = 187) reported the prevalence of specific disease(s), though a minority reported symptoms or lung function test (**Table 2**). Asthma was the most common disease studied (n = 144) followed by COPD (n = 112); 22 articles reported the prevalence of both asthma and COPD. Asthma COPD Overlap Syndrome (ACOS) was specifically explored in two recent surveys [40,41].

**Table 2:** Study outcomes

Outcome	Number, n (%)
Disease only	187 (67)
Combination of disease, symptom and/or lung function	57 (21)
Symptoms only	22 (8)
Lung function only	15 (5)

### Question 3. How was the socio-economic burden (from a societal or health care perspective) of asthma, COPD and other CRDs estimated in these surveys?

Burden (or impact) of respiratory disease was reported in only 33 publications. The most common variable was activity limitation (n = 22) followed by quality of life (n = 13), work absenteeism (n = 12) and psychosocial distress (n = 5). Our search strategy focussed on prevalence studies and although we included ‘cost’ as a word in the title or abstract, we did not use search terms for economic evaluation of the diseases. Health care utilisation, and health status were reported in 10 and 8 studies, respectively.

### Question 4. What strategies have been used to identify phenotypes of asthma and COPD, or to identify the causes of ‘other CRD’?

COPD was linked to either environmental factors (n = 60) or occupation (n = 32). Only five articles (four of which were published in the last 10 years) specifically used the term ‘phenotype’ when describing different classifications of asthma or COPD [41-45]. Articles that reported on ACOS (n = 2) were published in the last three years [40,41].

## DISCUSSION

### Summary of findings

We identified 281 publications reporting community surveys employing diverse methods for assessing the prevalence of CRDs in general populations in LMICs. The studies were conducted in 70 countries; nearly half were from Asia. Ten articles reported surveys with more than100 000 respondents; 101 studies had fewer than 1000 respondents. Surveys typically focussed on detecting either asthma (122 studies) or COPD (90 studies). 22 studies explicitly aimed to identify both conditions and very few detected ‘other CRDs’. Most studies used questionnaires derived from validated surveys (most commonly the ECRHS). Detection of asthma was typically based on symptoms or self-reported diagnosis. In contrast, COPD diagnosis was based on demonstration of obstruction on spirometry (without consideration of symptoms in 55%). The burden of CRD was reported in 33 publications, most commonly activity limitation. Only five recent articles used the term ‘phenotype’.

### Interpretation with reference to other literature

Prevalence estimates depend on the methods and diagnostic criteria used [46]. This was previously highlighted in a systematic review done in Europe [47] which showed that reported epidemiological estimates varied due to different methods including study designs. The most popular design used for studying disease prevalence in the community was household surveys. Postal surveys were not useful due to poor response rate [48-50], and one study found that the postal questionnaires were completed by persons other than the individual invited [48]. There was evidence that some surveys included quality measures, such as careful population-based sampling, standardised spirometry equipment, training and monitoring of spirometry technicians; over-reading of spirometry reports and strict protocols for questionnaire [16].

The studies in this review used a variety of operational definitions for asthma and COPD, employed a range of instruments, and modes of data collection although the increasing number of multi-country studies (eg, BOLD [16], PLATINO [51], PUMA [52], BREATHE [53]) have begun to standardize disease definitions. COPD guidelines are clear that a diagnosis of COPD relies on the demonstration of obstructive spirometry in a patient with chronic respiratory symptoms [54,55], yet 57 out of 112 studies in this review equated obstructive spirometry with COPD with no consideration of whether the patient had symptoms.

In contrast, the range of methods used to detect asthma reflects current discussions about the best way to confirm a diagnosis of asthma in clinical practice [56,57]. In LMICs, it has been suggested that the presence of asthma is better detected by having three components to the diagnosis, ie, presence of symptoms, reported physician-diagnosis, use of medication [26]. However, these approaches are often limited by inaccurate recall and recent studies have questioned the accuracy of physician diagnosis [58]. We found examples of all three of these approaches, but with little evidence of an emerging consensus, which makes it difficult to compare results across studies.

### Spirometry

In our review, 167 surveys conducted spirometry to define airway obstruction, but variation in the conduct of the test and interpretation of results means that the findings are not comparable.

The optimal threshold for diagnosing airflow obstruction has been hotly debated for some years [46] and guidelines make different recommendations [13,54,59]. Almost all the early surveys in our review, including multinational BOLD and PLATINO studies, used a fixed ratio of <70% as the diagnostic threshold for obstruction, aligned with global COPD guideline recommendations [16,54]. This however would underestimate the prevalence of COPD in young populations (for example, in the FRESH AIR study in Uganda where women exposed to biomass fuel since childhood had severe obstruction by the age of 35 [60]) and overestimate the prevalence of COPD in older populations [61-63]. The use of the LLN instead of a fixed criterion was advocated in 2004 [64], and in our review, studies conducted after 2008 started to use LLN, often reporting both. Use of LLN requires robust normal values from the relevant population which are not always available for LMIC countries [65], a challenge addressed in a study from Nigeria in which local normal values were derived from the non-smoking study population [66].

Discrepancies also arose because only two thirds of the studies used the recommended post-bronchodilator spirometry results, which could make considerable difference to the defined prevalence of airway obstruction [67]. A challenge, which is not yet reflected in the studies, is the recent change to the ATS/ERS standards requiring an inspiratory loop to assess for full inspiration to reduce the risk of mis-diagnosis of restrictive disorders [59]. This will incur the additional cost of providing filters to ensure safe practice in LMICs, many of which have endemic TB.

### Evidence gaps and implications for future surveys in LMICs

Although surveys (questionnaires and lung function) remain the most practical approach for estimating prevalence of CRD, our scoping review has identified several evidence gaps which should be considered in future CRDS surveys in LMICs:

- Many surveys focused on detecting one condition (asthma or COPD) only; a few identified both, but hardly any mentioned other CRDs.
- In line with current moves to personalised medicine [68], a few recent surveys have begun to consider phenotypes of asthma and COPD, but none used FeNO, which is gaining importance in phenotyping asthma [69].
- Algorithms for making a clinical diagnosis (as opposed to recording lung function) were often not well formulated, especially for asthma where the most predictive questions for the diagnosis are not clear.
- Although respiratory symptoms were assessed in most surveys, and many of the questionnaires used included impact on work and social activities, the impact on the quality of life of individuals, or the social and economic burden were rarely reported.

### Strengths and limitations

As this was a scoping review, it is useful for mapping common methodologies and identifying gaps in literature. However, no conclusions can be drawn on the quality of those studies. We focused on the methodologies used in community surveys, rather than analysing factors related to CRDs such as disease risks and prevalence rates.

Despite an inclusive search strategy and 36 872 hits, we may have missed some relevant studies. We did not include LILACS or Chinese language databases; however, our searches detected 30 and 42 studies conducted in South American countries (Argentina, Brazil, Chile, Colombia, Peru, Uruguay and Venezuela) and China respectively (Table S2 in the **Online Supplementary Document**). We did not impose a language limitation, planning to translate where possible. In the event, however, we were unable to arrange translation within our limited resources and 32 non-English papers were excluded. We had planned to check the Global Health Data Exchange website (http://ghdx.healthdata.org) for additional studies, but our checks for the four RESPIRE countries did not identify any new publications. We emailed thirteen authors to get full text articles, however only two authors responded.

In this review we aimed to scope surveys conducted in LMICs, but some multi-country surveys (eg, BOLD studies) took place in a number of high-income countries as well as several LMICs. Similarly, we were interested in surveys of CRD in adults, but some articles also included children. However, our interest was in the methodology used, which remained applicable. Even though we took steps to ensure that reviewers had at least 90% agreement in data extraction, individual reviewers’ interpretation of methodological framework could be a source of bias. We did not search for health economic evaluations, though we included ‘cost’ as a search term, which limits the conclusions we can draw about socio-economic impact. Studies from the same study population were reported as independent studies; it is possible that study populations and similar processes were duplicated.

## CONCLUSION

Accurate prevalence and disease burden information is important to understand the impact of CRDs on disability, quality of life, and to help influence public health care planning. Our scoping review identified several prevalence surveys for CRDs from a wide range of LMIC countries, but with substantial heterogeneity across the definitions, methodologies, instruments and types of outcomes used. Surveys remain the most practical approach for estimating the prevalence of CRD but there is a need to identify the most predictive questions for diagnosis asthma and to standardise diagnostic criteria. To reflect the true burden of CRDs in LMICs, future work should identify the wide range of conditions (not just asthma or COPD), and capture information on the burden of disease on an individual’s quality of life and the societal burden.

## Additional material

Online Supplementary Document
