# Supplementary material for: Chronic respiratory disease surveys in adults in low- and middle-income countries: A systematic scoping review of methodological approaches and outcomes
Source: J Glob Health. 2021 Jun 19;11:04026. doi: 10.7189/jogh.11.04026 (PMC8248510; doi:10.7189/jogh.11.04026)
Supplement: Online Supplementary Document [file jogh-11-04026-s001.pdf]

Chronic respiratory disease surveys in low/middle-income countries: A systematic scoping review of methodologies and outcomes

Appendix 1 Online Supplementary Document

Supplemental Table	Title
Table S1	MEDLINE search strategy
Table S2	Characteristics of Included Studies

Table S1: MEDLINE search strategy

#1	chronic respiratory disease*.ti,ab.
#2	(chronic obstructive pulmonary disease or obstructive airway* disease*).ti,ab.
#3	((tuberculosis or TB) adj1 (post or history or anterior)).ti,ab.
#4	Asthma.ti,ab.
#5	Asthma/
#6	Pulmonary disease, chronic obstructive/
#7	(prevalence or incidence or epidemiol* or survey or rapid assessment or situation assessment or situational assessment or rar or cohort or surveillance or screening).ti,ab.
#8	exp cohort studies/ or exp mass screening/ or exp population surveillance/
#9	epidemiologic methods/ or epidemiologic studies/ or sentinel surveillance/ or cross-sectional studies/ or public health surveillance/ or sentinel surveillance/ or epidemiological monitoring/ or epidemiologic studies/ or epidemiological methods/
#10	(cost or burden).ti,ab.
#11	africa/ or asia/ or caribbean/ or west indies/ or South america/ or latin america/ or central america/
#12	DEVELOPING COUNTRIES/
#13	((developing or less* developed or under developed or underdeveloped or middle income or low* income or underserved or under served or deprived or poor*) adj3 (countr* or nation* or population* or world)).mp.

#14	((developing or less* developed or under developed or underdeveloped or middle income or low* income) adj1 (economy or economies)).mp.
#15	(low* adj1 (GDP or GNP or gross domestic or gross national)).mp.
#16	(low adj3 middle adj3 countr*).mp.
#17	(LMIC or LMICs or third world or LAMI country or LAMI countries).mp.
#18	(transitional country or transitional countries).mp.
#19	(Afghanistan or Albania or Algeria or Angola or Antigua or Barbuda or Argentina or Armenia or Armenian or Aruba or Azerbaijan or Bahrain or Bangladesh or Barbados or Benin or Byelarus or Byelorussian or Belarus or Belorussian or Belorussia or Belize or Bhutan or Bolivia or Bosnia or Herzegovina or Hercegovina or Botswana or Brasil or Brazil or Bulgaria or Burkina Faso or Burkina Fasso or Upper Volta or Burundi or Urundi or Cambodia or Khmer Republic or Kampuchea or Cameroon or Cameroons or Cameron or Camerons or Cape Verde or Central African Republic or Chad or Chile or China or Colombia or Comoros or Comoro Islands or Comores or Mayotte or Congo or Zaire or Costa Rica or Cote d'Ivoire or Ivory Coast or Croatia or Cuba or Cyprus or Czechoslovakia or Czech Republic or Slovakia or Slovak Republic or Djibouti or French Somaliland or Dominica or Dominican Republic or East Timor or East Timur or Timor Leste or Ecuador or Egypt or United Arab Republic or El Salvador or Eritrea or Estonia or Ethiopia or Fiji or Gabon or Gabonese Republic or Gambia or Gaza or Georgia or Georgian or Ghana or Gold Coast or Greece or Grenada or Guatemala or Guinea or Guam or Guiana or Guyana or Haiti or Honduras or Hungary or India or Maldives or Indonesia or Iran or Iraq or Isle of Man or Jamaica or Jordan or Kazakhstan or Kazakh or Kenya or Kiribati or Korea or Kosovo or Kyrgyzstan or Kirghizia or Kyrgyz Republic or Kirghiz or Kirgizstan or Lao PDR or Laos or Latvia or Lebanon or Lesotho or Basutoland or Liberia or Libya or Lithuania or Macedonia or Madagascar or Malagasy Republic or Malaysia or Malaya or Malay or Sabah or Sarawak or Malawi or Nyasaland or Mali or Malta or Marshall Islands or Mauritania or Mauritius or Agalega Islands or Mexico or Micronesia or Middle East or Moldova or Moldovia or Moldovian or Mongolia or Montenegro or Morocco or Ifni or Mozambique or Myanmar or Myanma or Burma or Namibia or Nepal or Netherlands Antilles or New Caledonia or Nicaragua or Niger or Nigeria or Northern Mariana Islands or Oman or Muscat or Pakistan or Palau or Palestine or Panama or Paraguay or Peru or Philippines or Philipines or Phillipines or Phillippines or Poland or Portugal or Puerto Rico or Romania or Rumania or Roumania or Russia or Russian or Rwanda or Ruanda or Saint Kitts or St Kitts or Nevis or Saint Lucia or St Lucia or Saint Vincent or St Vincent or Grenadines or Samoa or Samoan Islands or Navigator Island or Navigator Islands or Sao

	Tome or Saudi Arabia or Senegal or Serbia or Montenegro or Seychelles or Sierra Leone or Slovenia or Sri Lanka or Ceylon or Solomon Islands or Somalia or Sudan or Suriname or Surinam or Swaziland or Syria or Tajikistan or Tadzhikistan or Tadjikistan or Tadzhik or Tanzania or Thailand or Togo or Togolese Republic or Tonga or Trinidad or Tobago or Tunisia or Turkey or Turkmenistan or Turkmen or Uganda or Ukraine or Uruguay or USSR or Soviet Union or Union of Soviet Socialist Republics or Uzbekistan or Uzbek or Vanuatu or New Hebrides or Venezuela or Vietnam or Viet Nam or West Bank or Yemen or Yugoslavia or Zambia or Zimbabwe or Rhodesia).ti,ab.
#20	or/1-6
#21	or/7-10
#22	or/11-19
#23	20 and 21 and 22
#24	limit 23 to (humans and year="1995 -Current")

Table S2: Characteristics of Included Studies

Author; year; country	Questionnaire	Study description	Population	Age (in years)	Number of participants (n); response rate (%)	Measured outcomes				
						Asthma	COPD	Other CRDs	Symp toms	Lung function test
Abbasi [1]; 2012; Pakistan	ATS-DLD-78-A	Cross-sectional house-to-house survey	General population	> 18	200 (Not available)	●			●	●
Abu Sham'a [2]; 2010; Palestine	ATS-DLD-78-A	Cross-sectional worksite survey	Farmers	22-77	250 (Not available)	●			●	
Aggarwal [3] 2006; India	IUATLD	Cross-sectional interviewer-assisted house-to-house survey	General population	> 15	73,605;100	●				
Agrawal [4]; 2012; India	NFHS-3	Secondary data analysis	General population	20-49	156,316;98	●				

Author; year; country	Questionnaire	Study description	Population	Age (in years)	Number of participants (n); response rate (%)	Measured outcomes				
						Asthma	COPD	Other CRDs	Symp toms	Lung function test
Agrawal [5]; 2013; India	NFHS-3	Secondary data analysis	General population	20-49	156,316;98	●				
Agrawal [6]; 2014; India	NFHS-3	Secondary data analysis	General population	15-54 (males), 15-49 (female)	117,719 (Not available)	●				
Ahasan [7]; 2000; Bangladesh	Self-designed	Cross-sectional worksite survey	Textile mill workers	18-52	210 (Not available)	●			●	
Akkurt [8]; 2003; Turkey	ECRHS	Cross-sectional survey	General population	> 20	5,448;93.2	●			●	
Akpinar-Elci [9], 2002; Turkey	ATS	Cross-sectional worksite survey	Hairdressers	Mean age: 25.2 ± 10.7	184;89.3	●				
Akpinar-Elci [10]; 2004; Turkey	ATS-DLD-78-A	Cross-sectional worksite survey	Florists	12-66	128;80.5	●			●	
Akpinar-Elci [11]; 2015; Grenada	ATS	Cross-sectional interviewer-assisted house-to-house survey	General population	> 18	225;80.1	●			●	
Akpinar-Elci [12]; 2017; Grenada	ATSDR, Occupational and Environmental Exposure History Questionnaire, ATS, Respiratory Disease Questionnaire, NHANES III, Respiratory Health and	Cross-sectional worksite survey	Nutmeg production workers	48.3 ± 8.1	92 (Not available)	●			●	

Author; year; country	Questionnaire	Study description	Population	Age (in years)	Number of participants (n); response rate (%)	Measured outcomes				
						Asthma	COPD	Other CRDs	Symp toms	Lung function test
	Disease Questionnaire									
Alam [13]; 2015; Bangladesh	Self-designed	Cross-sectional interviewer-assisted house-to-house survey	General population	> 40	3,744;99.6		●			
Amaral [14]; 2018; 29 Countries	BOLD	Cross-sectional interviewer-assisted house-to-house survey	General population	> 40	18,554 (Not available)					●
Amiri [15]; 2014; Iran	ECRHS	Cross-sectional interviewer-assisted house-to-house survey	General population	NA	857 (Not available)	●				
Andreeva [16]; 2015; Russia	ATS-DLD-78-A and IPCRG Questionnaire	Prospective cohort study	General population	35-70	3,133 (Not available)	●	●			
Andreeva [17]; 2016; Russia	ATS-DLD-78-A	Cross-sectional healthcare facility survey	General population	35-70	2,974 (Not available)		●			
Arias [18]; 2018; Argentina	ECRHS	Cross-sectional computer-assisted telephone survey	General population	20-44	1,521; 51.4	●				
Arora [19];2018; India	Not reported	Cross-sectional interviewer-assisted house-to-house survey	Women	18-59	299 (Not available)					●
Arslan [20]; 2013; Turkey	BOLD	Cross-sectional healthcare facility survey	General population	> 40	946 (Not available)		●			
Athavale [21]; 2017; India	IUATLD	Cross-sectional self-administered house-to-house survey	General population	<20 - 80	1,006 (Not available)	●				
Baatjies [22]; 2009; South Africa	ECRHS	Cross-sectional worksite survey	Bakery Workers	Mean age:32	517 (Not available)	●			●	●

Author; year; country	Questionnaire	Study description	Population	Age (in years)	Number of participants (n); response rate (%)	Measured outcomes				
						Asthma	COPD	Other CRDs	Symp toms	Lung function test
Badway [23]; 2016; Egypt	CAT	Cross-sectional interviewer-assisted house-to-house survey	General population	> 40	2,400;96		●			
Balcan [24]; 2018; Turkey	Self-designed	Cross-sectional healthcare facility survey	clinic/hospital attendees / staff	> 18	424 (Not available)					●
Banda [25]; 2017; Malawi	S1 questionnaire	Cross-sectional interviewer-assisted house-to-house survey	General population	15-99	15,795;95.1				●	
Barbieri [26]; 2006; Brazil	ECRHS, IPAQ	Prospective cohort study	General population	23-25	2,063 (Not available)	●				
Baris [27]; 2011; Turkey	ECRHS	Cross-sectional worksite survey	Teachers	Mean age: 38.9 ± 8.9	685 (Not available)		●			
Boskabady [28]; 2002; Iran	Self-designed	Cross-sectional interviewer-assisted house-to-house survey	General population	≥ 20	5,579 (Not available)	●				
Budhathoki [29]; 2016; Nepal	Self-designed	Cross-sectional worksite survey	Welders	≤ 49	300 (Not available)	●			●	
Buist [30]; 2008; China, Turkey, Austria, South Africa, Iceland, Germany, Poland, Norway, Canada, USA, Philippines, Australia	BOLD	Cross-sectional interviewer-assisted house-to-house survey	General population	> 40	9,425 (Not available)		●			
Caballero [31]; 2008; Colombia	Ferris Respiratory Symptoms and	Cross-sectional interviewer-assisted house-to-house survey	General population	> 40	5,539; Not available		●			

Author; year; country	Questionnaire	Study description	Population	Age (in years)	Number of participants (n); response rate (%)	Measured outcomes				
						Asthma	COPD	Other CRDs	Symp toms	Lung function test
	Risk Factors Questionnaire									
Caban-Martinez [32]; 2012; Dominican Republic	CDC-Behavioral Risk Factor Surveillance System (BRFSS)	Cross-sectional self-administered community (health fair) survey	General population	18-79	117; 49	●				
Caldeira [33]; 2006; Brazil	Symptoms and Risk Factors Questionnaire	Prospective cohort study	Young adults	23-25	1,922; 93.1	●				
Cardoso [34]; 2012; Brazil	ISAAC	Cross-sectional interviewer-assisted house-to-house survey	General population	6-50	427; Not available	●				
Celedon [35]; 2000; China	ATS-DLD	Cross-sectional interviewer-assisted house-to-house survey	Family with known asthma members	No age limit (7,671 offspring; 5,324 parents)	12,995 subjects in 2,756 families; Not available	●	●			
Celik [36]; 1999; Turkey	ECRHS	Cross-sectional interviewer-assisted survey	General population	18-72	1,056;81.2	●			●	
Chan [37]; 2015; Malaysia	IPAQ and WHO STEPS	Cross-sectional interviewer-assisted house-to-house survey	General population	> 18	18,184;99.7	●				
Chan-Yeung [38]; 2002; China	IUATLD	Cross-sectional interviewer-assisted house-to-house survey	General population	20-44	22,528; Not available	●			●	
Chattopadhyay [39]; 2014; India	St. George's Questionnaire	Cross-sectional worksite survey	Coal-based sponge iron plant workers	Mean age: 35±8	258; 100				●	

Author; year; country	Questionnaire	Study description	Population	Age (in years)	Number of participants (n); response rate (%)	Measured outcomes				
						Asthma	COPD	Other CRDs	Symp toms	Lung function test
Chhabra [40]; 2001; India	ATS-DLD-78-A, BMRC and the National Heart, Lung and Blood Institute (NHLBI-USA)	Cross-sectional interviewer-assisted house-to-house survey	General population	> 18	4,171; Not available	●	●	●	●	
Chien [41]; 2002; Vietnam	ATS	Cross-sectional worksite survey	Refractory brick facility workers	24-49	158; Not available			●		
Ching [42]; 2014; Malaysia	Canadian case finding questionnaire	Cross-sectional healthcare facility survey	Smokers	> 40	416; Not available		●			●
Chkhaidze [43]; 2009; Georgia	Self-designed	Cross-sectional healthcare facility survey	clinic/hospital attendees / staffs	≥ 5	3,646; Not available	●		●		
Chowgule [44]; 1998; India	IUATLD	Cross-sectional interviewer-assisted house-to-house survey	General population	20-44	2,213;81	●			●	
Choy [45]; 2002; China	Self-designed	Cross-sectional interviewer-assisted house-to-house survey	Elderly	> 70	2,032;59.7	●			●	
Chuaychoo [46]; 2003; Thailand	Self-designed	Not available	General population	> 60	3,094;99.1		●			
Chuchalin [47]; 2014; Russia	GARD	Cross-sectional interviewer-assisted house-to-house survey	General population	> 18	7,164; Not available	●	●	●	●	
Daldoul [48]; 2013; Tunisia	BOLD	Cross-sectional interviewer-assisted house-to-house survey	General population	> 40	717;88.8		●			

Author; year; country	Questionnaire	Study description	Population	Age (in years)	Number of participants (n); response rate (%)	Measured outcomes				
						Asthma	COPD	Other CRDs	Symp toms	Lung function test
Davey [49]; 2005; Ethiopia	IUATLD	Cross-sectional interviewer-assisted house-to-house survey	General population	> 5	3,485 urban 4,164 rural ; 81 urban and 91 rural	●				
de Fátima Macaira [50], 2007; Brazil	ECRHS, ISAAC and MRC questionnaire	Cross-sectional worksite survey	Non-domestic cleaners	Mean age: 35(women), 30.7(men)	341; Not available	●	●			
de Oca [51], 2012; Brazil, Chile,Mexico, Uruguay, Venezuela	PLATINO, SF-12	Cross-sectional interviewer-assisted house-to-house survey	General population	> 40	5,571; Not available		●	●	●	
de Oca [52], 2017; Latin America	PLATINO	Cross-sectional interviewer-assisted house-to-house survey	General population	> 40	1,743; Not available	●	●			
de Sousa [53] 2011; Brazil	2008 Health Survey of the City of São Paulo (ISA-Capital 2008)	Cross-sectional interviewer-assisted house-to-house survey	General population	≥ 40	1,441;77.5		●			
de Souza [54] , 2010; Brazil	Self-designed	Cross-sectional worksite survey	Charcoal production workers	Mean age: 46.5 ± 13.3	67;100	●	●		●	
Dejsomritrutai [55]; 2006; Thailand	IUATLD	Cross-sectional healthcare facility survey	General population	20-44	3,454; 46.9	●				

Author; year; country	Questionnaire	Study description	Population	Age (in years)	Number of participants (n); response rate (%)	Measured outcomes				
						Asthma	COPD	Other CRDs	Symp toms	Lung function test
Dejsomritrutai [56]; 2009; Thailand	Self-designed	Cross-sectional house-to-house survey	General population	20-44	268; Not available	●				
Denguezli [57]; 2016; Tunisia	BOLD	Cross-sectional interviewer-assisted house-to-house survey	Non-smokers	> 40	717;90		●			
Dennis [58]; 2012; Colombia	ISAAC	Cross-sectional self-administered survey	General population	Jan-59	5,978; Not available	●				
Desalu [59]; 2009; Nigeria	ECRHS	Cross-sectional interviewer-assisted house-to-house survey	General population	18-65	805;100	●				
Desalu [60]; 2011; Nigeria	ECSC	Cross-sectional interviewer-assisted house-to-house survey	General population	> 35	391;86.7				●	
Deschamps [61]; 1998; Senegal	ATS	Cross-sectional worksite survey	Workers Exposed to Isocyanytes	Mean age: 39.25	96; Not available	●		●	●	
Deveci [62]; 2011; Turkey	BOLD	Cross-sectional interviewer-assisted house-to-house survey	General population	> 18	1,206;94.9		●			
Díaz [63]; 2007; Guatemala	ISAAC, IUATLD and MRC questionnaire	Cross-sectional interviewer-assisted house-to-house survey	Women	15-50	350; Not available				●	
Ding [64]; 2012; China	Self-designed	Cross-sectional interviewer-assisted house-to-house survey	General population	> 2	13,050; Not available	●				
Ding [65]; 2015; China	Self-designed	Cross-sectional interviewer-assisted house-to-house survey	General population	> 40	5,463; Not available		●			

Author; year; country	Questionnaire	Study description	Population	Age (in years)	Number of participants (n); response rate (%)	Measured outcomes				
						Asthma	COPD	Other CRDs	Symp toms	Lung function test
Ding [66]; 2018; China	BOLD	Cross-sectional interviewer-assisted house-to-house survey	General population	> 40	5,637; 100		●			
Dong [67], 2013; China	ATS	Self-administered community survey	Women	23-49	30,780; 89	●		●		
Dutta [68]; 2015; India	IUATLD	Cross-sectional interviewer-assisted house-to-house survey	Women	> 40	1,650; Not available			●		
Echazarreta [69]; 2018; Argentina	CAT, mMRC questionnaire	Cross-sectional interviewer-assisted house-to-house survey	General population	≥ 40	4,019; 87		●			
Ehrlich [70]; 2004; South Africa	South African Demographic and Health Survey	Cross-sectional interviewer-assisted house-to-house survey	General population	≥ 15	13,722; Not available				●	
El Hasnaoui [71], 2012	Confronting COPD questionnaire, CAT	Cross-sectional telephone survey	General population	> 40	62,086; 74		●			
El Rhazi [72] , 2016; Morocco	BOLD	Cross-sectional interviewer-assisted house-to-house survey	General population	> 40	966; 96		●			
Erhabor [73]; 2006; Nigeria	IUATLD and MRC questionnaire	Cross-sectional self-administered survey	University students	15-35	903; 90	●			●	
Erhabor [74]; 2016; Nigeria	ECRHS	Cross-sectional self-administered university survey	University students and staff	> 15	Students:2,37 2 Staff:455; Students: 86.3 Staff: 32	●				

Author; year; country	Questionnaire	Study description	Population	Age (in years)	Number of participants (n); response rate (%)	Measured outcomes				
						Asthma	COPD	Other CRDs	Symp toms	Lung function test
Fazlollahi [75]; 2018; Iran	ECRHS	Cross-sectional interviewer-assisted house-to-house survey	General population	20-44	24,344; Not available	●				
Fereidouni [76]; 2009; Iran	ECRHS	Community survey	General population	Mean age: 25	1,230; 88	●				
Franco-Marina [77]; 2014; Mexico	PLATINO	Cross-sectional interviewer-assisted house-to-house survey	General population	≥ 40	659; Not available		●			
Gathuru [78]; 2002; Nigeria	ATS	Cross-sectional worksite survey	Civil servants	30-69	410; Not available				●	●
Ghasemkhani [79]; 2006; Iran	modified MRC questionnaire	Cross-sectional worksite survey	Industrial workers	19-70	880; Not available				●	
Gizaw [80]; 2016; Ethiopia	MRC questionnaire	Cross-sectional worksite survey	Cement factory workers	18-60	404; 95				●	
Golshan [81]; 2002; Iran	Self-designed	Cross-sectional healthcare facility survey	General population	13-37	994; 88	●				
Golshan [82]; 2001; Iran	Self-designed	Cross-sectional healthcare facility survey	General population	> 35	4,636; 81		●		●	
Golshan [83]; 2002; Iran	Self-designed	Cross-sectional survey	General population	1-80	994; 88				●	
Golshan [84]; 2011; Iran	European Compendium of Respiratory Standard	Cross-sectional interviewer-assisted house-to-house survey	General population	≥ 40	1,308; 59		●			
Gonzalez-Garcia [85]; 2015; Colombia	ATS-DLD-78-A	Cross-sectional interviewer-assisted house-to-house survey	General population	40-93	5,539; Not available	●				

Author; year; country	Questionnaire	Study description	Population	Age (in years)	Number of participants (n); response rate (%)	Measured outcomes				
						Asthma	COPD	Other CRDs	Symp toms	Lung function test
Gorgieva [86]; 2010; Kosovo	Self-designed	Cross-sectional interviewer-assisted house-to-house survey	General population	> 18	423; Not available		●			
Gourgoulianis [87]; 2000; Greece	Not reported	Cross-sectional self-administered house-to-house survey	General population	> 55	569; Not available				●	
Guddattu [88]; 2010; India	Not reported	Secondary data analysis	Women	15-49	124,39; Not available	●				
Gunen [89]; 2008; Turkey	BOLD	Cross-sectional interviewer-assisted house-to-house survey	General population	> 18	1,160; 93		●			
Gupta [90]; 2006; India	IUATLD	Cross-sectional house-to-house survey	General population	> 15	62,109; Not available	●				
Gupta [91]; 2016; India	IUATLD	Cross-sectional survey	General population	> 60	1,522; Not available	●	●			
Gupta [92]; 2017; India	Self-designed	Community survey	General population	Not available	73; Not available	●	●		●	
Hamatui [93], 2017; Namibia	Adapted questionnaire from another study	Cross-sectional self-administered house-to-house survey	General population	> 18	107; Not available	●	●		●	
Hamzaçebi [94]; 2006; Turkey	ECRHS	Cross-sectional healthcare facility survey	General population	15-80	1,916; 89	●			●	
Han [95]; 2018; Puerto Rico	Self-designed	Cross-sectional interviewer-assisted house-to-house survey	General population	18-64	3,049; 84	●	●			
Horner [96], 2017; pooled data from participating	BOLD, EPISCAN, PLATINO, and PREPOCOL questionnaire	Cross-sectional interviewer-assisted house-to-house survey	General population	Mean age: 56.1 ±11.3	30,874; Not available		●			

Author; year; country	Questionnaire	Study description	Population	Age (in years)	Number of participants (n); response rate (%)	Measured outcomes				
						Asthma	COPD	Other CRDs	Symp toms	Lung function test
countries (44 sites)										
Huang [97]; 2013; China	Self-designed	Prospective cohort survey	General population	Not available	49,383; 95		●			
Idolor [98]; 2011; Philippines	BOLD	Cross-sectional interviewer-assisted house-to-house survey	General population	> 40	991; Not available		●			
Ishtiaq [99]; 2014; Pakistan	Self-designed	Cross-sectional worksite survey	Coal Miners	Mean age: 30	400; Not available			●		
Jaganath [100]; 2015; Peru	Not reported	Cross-sectional interviewer-assisted house-to-house survey	General population	> 35	3601; 83		●			
Jeebhay [101]; 2008; South Africa	ECRHS	Cross-sectional worksite survey	Fish processing workers	Mean age: 36± 11	594; Not available	●				
Jie [102]; 2013; China	ECRHS	Cross-sectional self-administered house-to-house survey	General population	> 18	1,207; 95	●			●	
Jie [103]; 2016; China	Self-designed	Cross-sectional self-administered house-to-house survey	General population	> 18	610; Not available					●
Jie [104]; 2016; China	ECRHS II	Cross-sectional self-administered house-to-house survey	General population	> 18	610; 100	●				
Jindal [105]; 2000; India	IUATLD	Cross-sectional healthcare facility survey	General population	> 18	1,283; 86	●				
Jindal [106]; 2006; India	Self-designed	Cross-sectional interviewer-assisted house-to-house survey	General population	> 35	35,295; Not available		●			

Author; year; country	Questionnaire	Study description	Population	Age (in years)	Number of participants (n); response rate (%)	Measured outcomes				
						Asthma	COPD	Other CRDs	Symp toms	Lung function test
Jindal [107]; 2012; India	IUALTD	Cross-sectional house-to-house survey	General population	> 15	169,575; 96	●		●	●	
Johnson [108], 2011; India	Not reported	Cross-sectional interviewer-assisted house-to-house survey	Rural women	≥ 30	900; Not available		●			
Kahwa [109], 2010; Jamaica	ECRHS II, ISAAC II	Cross-sectional interviewer-assisted house-to-house survey	General population	> 18 (Adult) 2-17 (children)	2163; 89 (adult) 80 (children)	●				
Kavishe [110], 2015; Tanzania and Uganda	WHOSTEPS	Cross-sectional interviewer-assisted house-to-house survey	General population	> 18	1,095 (Tanzania) 916 (Uganda); 80(Tanzania) & 79 (Uganda)		●			
Ko [111], 2008; China	ATS	Cross-sectional healthcare facility survey	Elderly	> 60	1,008; 88		●			
Konuk [112]; 2017; Turkey	Self-designed	Community survey	General population	> 35	500; Not available		●			
Konuk [113]; 2017; Turkey	Not reported	Cross-sectional healthcare facility survey	clinic/hospital attendees/ staff	> 30	1,000; Not available		●			
Koul [114]; 2016; India	BOLD	Cross-sectional interviewer-assisted house-to-house survey	General population	> 40	757; 87	●	●			
Kourlaba [115]; 2018; Greece	ACT, EQ-5D	Cross-sectional telephone survey	General population	> 18	3,946; 99	●				
Kumar [116]; 2017; India	IUATLD	Cross-sectional interviewer-assisted house-to-house survey	General population	18-70	3,194; Not available	●				

Author; year; country	Questionnaire	Study description	Population	Age (in years)	Number of participants (n); response rate (%)	Measured outcomes				
						Asthma	COPD	Other CRDs	Symp toms	Lung function test
Kurmi [117]; 2013; Nepal	Not reported	Cross-sectional interviewer-assisted house-to-house survey	General population	> 16	1,392; Not available					●
Kurmi [118]; 2015; China	CKB questionnaire	Cross-sectional healthcare facility survey	General population	35-74	512,891; 30		●	●		
Kurt [119]; 2011; Turkey	Self-designed	Cross-sectional healthcare facility survey	General population	> 18	1,047; Not available	●				
Lai [120]; 1995; China	Adapted questionnaire from another study	Cross-sectional interviewer-assisted survey	Elderly	> 70	2,032; 60	●				
Lai [121]; 2013; China	Self-designed	Prospective study, healthcare facility survey	General population	> 15	704; Not available	●			●	
Lam [122]; 2010; China	Not available	Prospective cohort study	Elderly	> 50	8,066; Not available					●
Lâm [123] , 2011; Vietnam	Swedish OLIN	Community survey	General population	21-70	5,782; 83	●		●		
Lam [124]; 2012; China	MRC questionnaire	Prospective cohort study	General population	> 50	18,787; Not available	●	●			
Lam [125]; 2014; Vietnam	GA2LEN, Swedish OLIN	Cross-sectional interviewer-assisted house-to-house survey	General population	23-72	565; 83		●			
Lamprecht [126]; 2011; China, Turkey, Austria, South Africa, Iceland, Germany, Poland, Norway, Canada, USA, Philippines,	BOLD	Cross-sectional interviewer-assisted house-to-house survey	Non-smokers	> 40	10,000; Not available		●			

Author; year; country	Questionnaire	Study description	Population	Age (in years)	Number of participants (n); response rate (%)	Measured outcomes				
						Asthma	COPD	Other CRDs	Symp toms	Lung function test
Australia, UK and Sweden										
Laniado-Laborin [127]; 2011; Mexico	PLATINO	Cross-sectional healthcare facility survey	Patients at clinic with risk factors	> 40	2,293; 98		●			
Laraqui [128]; 2018; Morocco	ECSC, BMRC questionnaire, ATS and WHO	Cross-sectional worksite survey	Fisherman	> 20	924; 30	●	●		●	
Lim [129]; 2002; Malaysia	BMRC questionnaire	Cross-sectional worksite survey	Cement workers	> 15	1,164; Not available	●		●		
Lin [130]; 2017; Romania	ISAAC	Cross-sectional worksite survey	Teachers	Not available	104; Not available	●				
Lin [131]; 2018; China	CARE	Cross-sectional interviewer-assisted house-to-house survey	General population	≥ 40	164,215; Not available	●				
Liu [132]; 2007; China	Not reported	Cross-sectional healthcare facility survey	General population	> 40	3,286; 77		●			
Liu [133]; 2015; China	BOLD	Cross-sectional healthcare facility survey	Greenhouse farmers	<40 to >60	5,420; 92		●			
Liu [134]; 2017; China	BOLD	Cross-sectional healthcare facility survey	General population	> 20	5,993; 84		●			
Loh [135]; 2016; Malaysia	BOLD	Cross-sectional interviewer-assisted house-to-house survey	General population	> 40	663; 59		●			
Lopez Varela [136]; 2013; Latin America	PLATINO, SF-12	Cross-sectional interviewer-assisted house-to-house survey	General population	> 40	5,314; Not available		●			
Lopez Varela [137]; 2016; Argentina,	PLATINO	Cross-sectional healthcare facility survey	General population	> 40	1,540; Not available		●			●

Author; year; country	Questionnaire	Study description	Population	Age (in years)	Number of participants (n); response rate (%)	Measured outcomes				
						Asthma	COPD	Other CRDs	Symp toms	Lung function test
Colombia, Uruguay, Venezuela										
Lu [138]; 2010; China	BOLD	Cross-sectional interviewer-assisted house-to-house survey	General population	> 40	20,245; 79		●			
Luenam [139]; 2018; Thailand	National Socioeconomics Survey	Secondary data analysis	General population	mean age 43.27 ± 17.37 and 44.38 ± 18.16	33,945; Not available		●	●		
Mackenney [140]; 2005; Chile	ECRHS	Cross-sectional community survey follow-up study	General population	22-28	1,232; Not available	●				
Magitta [141]; 2018; Tanzania	BOLD	Cross-sectional interviewer-assisted house-to-house survey	General population	> 35	496; 57		●			
MAHESH [142]; 2009	Self-designed	Cross-sectional interviewer-assisted house-to-house survey	General population	>40	900 (2nd stage); 99.5		●			
Mahesh [143]; 2011; India	BOLD	Cross-sectional interviewer-assisted house-to-house survey	General population	> 31	4,333; Not available				●	
Mahesh [144]; 2014; India	BOLD	Cross-sectional interviewer-assisted house-to-house survey	Men	> 30	4,504; Not available		●		●	
Mamane [145]; 2016; Niger	ISAAC	Cross-sectional interviewer-assisted house-to-house survey	People living on farms	>7	471 adults and 229 children; Not available	●	●		●	

Author; year; country	Questionnaire	Study description	Population	Age (in years)	Number of participants (n); response rate (%)	Measured outcomes				
						Asthma	COPD	Other CRDs	Symp toms	Lung function test
Mannino [146]; 2012; China, Turkey, Austria, South Africa, Iceland, Germany, Poland, Norway, Canada, USA, Philippines, Australia, UK and Sweden	BOLD	Cross-sectional interviewer-assisted house-to-house survey	General population	> 40	9,762; Not available					●
Maranetra [147]; 2002; Thailand	Self-designed	Community survey	Elderly	> 60	3,094; Not available		●			
Maranetra [148]; 2003; Thailand	Not reported	Cross-sectional interviewer-assisted house-to-house survey	Elderly	> 60	3,094; Not available		●			
Masoompour [149]; 2018; Iran	ECRHS	Cross-sectional interviewer-assisted house-to-house survey	General population	20-60	4,582; 96	●				
Mberikunashe [150]; 2010; Zimbabwe	Self-designed	Cross-sectional worksite survey	Textile Industry Workers	Median age: 40	194; Not available					●
Megjhi [151]; 2016; Malawi	BOLD	Cross-sectional interviewer-assisted house-to-house survey	General population	> 18	1,469; 74		●			
Mejza [152]; 2017; Malawi, USA, Kyrgyzstan, UK, Saudi Arabia, Norway, Tunisia, South Africa,	BOLD	Cross-sectional healthcare facility survey	General population	> 40	23,855; Not available				●	

Author; year; country	Questionnaire	Study description	Population	Age (in years)	Number of participants (n); response rate (%)	Measured outcomes				
						Asthma	COPD	Other CRDs	Symp toms	Lung function test
Morocco, Sweden, Germany, India, Portugal , China, Poland, Iceland, Canada, Turkey, Kyrgyzstan, Austria, Philippines, Estonia, Sri Lanka, Australia, Netherlands, Algeria, Albania , India, Malaysia , India , Benin, Nigeria										
Meneghini [153]; 2017; Brazil	ECRHS	Cross-sectional healthcare facility survey	General population	23-25	1,922; Not available	●				
Menezes [154]; 2004; Brazil	ATS-DLD-78-A	Cross-sectional self-administered house-to-house survey	General population	40-69	1,046; 82 (subsample)		●			
Menezes [155]; 2005; Brazil	PLATINO	Cross-sectional interviewer-assisted house-to-house survey	General population	> 40	963; 85		●			
Menezes [156]; 2005; Brazil, Chile, Mexico, Uruguay, Venezuela	PLATINO	Cross-sectional interviewer-assisted house-to-house survey	General population	> 40	Brazil 1,000; 87. Chile 1,208, 82, Mexico: 1,063, 73, Uruguay:943,		●			

Author; year; country	Questionnaire	Study description	Population	Age (in years)	Number of participants (n); response rate (%)	Measured outcomes				
						Asthma	COPD	Other CRDs	Symp toms	Lung function test
					85, Venezuela:1357; 89					
Menezes [157]; 2008; Brazil	ISAAC	Prospective cohort study	General population	> 20	4,297; Not available				●	
Menezes [158]; 2008; Latin America	PLATINO	Cross-sectional interviewer-assisted house-to-house survey	General population	> 40	Not available		●			
Menezes [159]; 2015; Brazil	National survey	Cross-sectional technology assisted house-to-house survey	General population	≥ 18	60,202; Not available	●				
Menezes [160]; 2014; Latin America	PLATINO, SF-12, Fagerstrom scale, Baecke questionnaire and Beck Inventory Depression	Prospective cohort study	General population	> 40	885 (Montivedio), 1,173 (Santiago), 963(Sao Paola); 86 of patients in Montevideo, 85 in Santiago and 78 in São Paulo.		●			
Menezes [161]; 2017; Latin America	PLATINO	Cross-sectional healthcare facility survey	General population	> 40	524 in FU study; Not available	●			●	
Meren [162]; 2001; Estonia	Swedish OLIN, IUALTD, ECRHS	Postal survey	General population	15-64	17,525;78	●			●	
Miele [163]; 2016; Peru	WHO STEPS	Cross-sectional house-to-house survey	General population	> 35	2,946; Not available			●		

Author; year; country	Questionnaire	Study description	Population	Age (in years)	Number of participants (n); response rate (%)	Measured outcomes				
						Asthma	COPD	Other CRDs	Symp toms	Lung function test
Milenkovic [164]; 2011; Serbia	ECRHS	Postal survey	General population	20-80	5,079;58	●			●	
Minas [165]; 2010; Greece	self-designed	Cross-sectional healthcare facility survey	General population	> 30	1,526; Not available		●			
Miszkurka [166]; 2012; Burkina Faso	World Health Survey	Secondary data analysis	General population	> 18	4,822;98	●				
Mohammadi [167]; 2016; Iran	ECRHS	Cross-sectional self-administered university survey	University students	17-35	1,019;89	●				
Moreira [168]; 2015; Brazil	PLATINO	Prospective cohort study, interviewer-assisted, house-to-house	General population	> 40	613;61		●			
Morgan [169]; 2018; Uganda	modified World Health Survey, modified ECRHS and modified ACQ	Cross-sectional interviewer-assisted house-to-house survey	General population	> 35	1,769; Not available	●				
Mountjoy [170]; 2015; multiple countries	Not available	Cross-sectional survey	Athletes	Not available	1,468;81	●				
Mungan [171]; 2018; Egypt, Turkey, Kuwait, Saudi Arabia, UAE	ACT, EQ-5D	Cross-sectional telephone survey	General population	> 18	939; 44	●				
Musafiri [172]; 2011; Rwanda	ATS-DLD-78-A and ECRHS II	Cross-sectional interviewer-assisted healthcare facility survey	General population	15-80	1,920; 9	●	●		●	
Nafees [173]; 2013; Pakistan	ATS-DLD-78-A	Cross-sectional worksite survey	Men	> 18	37296	●		●	●	●

Author; year; country	Questionnaire	Study description	Population	Age (in years)	Number of participants (n); response rate (%)	Measured outcomes				
						Asthma	COPD	Other CRDs	Symp toms	Lung function test
Nafees [174]; 2016; Pakistan	ATS-DLD-78-A and WHO Respiratory questionnaire	Cross-sectional worksite survey	Textile workers	> 18	372; Not available		●	●	●	
Nafti [175]; 2009; Algeria, Morocco, Tunisia	ACT	Cross-sectional telephone survey	General population	Mean age: 40.3±17.1, 6.5±16.8, 44.3±20.3	62781;67	●				
Nakao [176]; 2017; Mongolia	Not reported	Cross-sectional healthcare facility survey	Hospital attendees	40-79	746;72	●	●			
Ngui [177]; 2011; Malaysia	IUATLD	Cross-sectional interviewer-assisted house-to-house survey	Aborigines	> 1	716; 61	●				
Nguyen Viet [178]; 2015; Indonesia, Vietnam	ATS-DLD-78-A, CAT, WHO questionnaire for assessment of indoor biomass burning	Cross-sectional interviewer-assisted house-to-house survey	General population	> 40	1,506 (756 Indonesia, 750 Vietnam); Not available		●		●	
Nriagu [179]; 1999; South Africa	WHO survey for asthma studies	Cross-sectional interviewer-assisted house-to-house survey	General population	adult >18 children <17	1,060 (693 adults, 367 children); Not available	●			●	
Nugmanova [180]; 2018; Ukraine, Kazakhstan, Azerbaijan	ATS Respiratory Symptoms Questionnaire, Allergic Rhinitis Questionnaire, Alcohol Intake, Tobacco	Cross-sectional interviewer-assisted house-to-house survey	General population	> 18	2,842; Not available	●			●	

Author; year; country	Questionnaire	Study description	Population	Age (in years)	Number of participants (n); response rate (%)	Measured outcomes				
						Asthma	COPD	Other CRDs	Symp toms	Lung function test
	Smoking Questions									
Nwibo [181]; 2012; Nigeria	Self-designed	Cross-sectional worksite survey	Quarry workers	10 - 60	403; Not available				●	
Obaseki [182]; 2014; Nigeria	ECRHS	Cross-sectional interviewer-assisted house-to-house survey	General population	> 15	2,310; 64	●				
Obaseki [183]; 2016; Nigeria	BOLD	Cross-sectional house-to-house survey	General population	> 40	1,169;76					●
Obaseki [184]; 2017; Nigeria	BOLD	Cross-sectional interviewer-assisted house-to-house survey	General population	> 40	1,147; Not available			●	●	●
Obaseki [185]; 2017; Nigeria	BOLD	Cross-sectional interviewer-assisted house-to-house survey	General population	> 40	1,169; 76					●
Obel [186]; 2017; Democratic Republic of Congo	ISAAC, ECRHS	Cross-sectional interviewer-assisted house-to-house survey	General population	> 18	1,088;83	●				
Ornek [187]; 2015; Turkey	BOLD	Cross-sectional interviewer-assisted house-to-house survey	General population	> 18	611; Not available		●			
Ory [188]; 1997; India	Not available	Cross-sectional worksite survey	Tannery workers	Mean age: 33	418; 100	●				
Ozdemir [189]; 2000; Turkey	ECRHS	Cross-sectional self-administered university survey	University students	Mean age: 20	1,515; 95	●				
Padhi [190]; 2008; India	IUATLD	Cross-sectional interviewer-assisted house-to-house survey	General population	20-40	750; Not available	●			●	

Author; year; country	Questionnaire	Study description	Population	Age (in years)	Number of participants (n); response rate (%)	Measured outcomes				
						Asthma	COPD	Other CRDs	Symp toms	Lung function test
Pan [191]; 2017; China	IPAQ-C	Prospective cohort study	General population	> 50	16,186 (From 30430); 53					●
Papageorgiou [192]; 1997; Greece	ECRHS	Postal survey	General population	20-44	3,325;79	●			●	
Parasuramalu [193]; 2010; India	IUATLD and Standard of Living Index (SLI)	Cross-sectional interviewer-assisted house-to-house survey	General population	18-70	3,194; Not available	●				
Parasuramalu [194]; 2014; India	Adapted questionnaire from another study	Cross-sectional interviewer-assisted house-to-house survey	General population	> 35	1,400; Not available		●			
Pefura-Yone [195]; 2015; Cameroon	ISAAC, ECRHS II	Cross-sectional interviewer-assisted house-to-house survey	General population	> 19	2,304;93	●				
Perez-Padilla [196]; 2012; Uruguay, Brazil, Chile, Venezuala, Mexico	PLATINO, SF-12	Cross-sectional interviewer-assisted house-to-house survey	Never-smokers	> 40	5,315; Not available		●			
Pothirat [197]; 2015; Thailand	ECRHS	Cross-sectional healthcare facility survey	General population	> 40	867; Not available		●			
Pothirat [198]; 2016; Thailand	ECRHS and mMRC	Cross-sectional interviewer-assisted house-to-house survey	General population	> 40	574; Not available	●	●			
Priftanji [199]; 1999, Albania	ECRHS	Cross-sectional healthcare facility survey	general population	20-44	2,653; Not available	●			●	
Quansah [200]; 2016; Ghana	Adapted questionnaire	Cross-sectional worksite survey	Farmers	> 18	300; Not available				●	

Author; year; country	Questionnaire	Study description	Population	Age (in years)	Number of participants (n); response rate (%)	Measured outcomes				
						Asthma	COPD	Other CRDs	Symp toms	Lung function test
	from another study									
Queiroz [201]; 2012; Brazil	modified PLATINO	Cross-sectional healthcare facility survey	clinic/hospital attendees / staff	> 40	200; Not available		●			
Rabahi [202]; 2015; Brazil	Not mentioned	Cross-sectional healthcare facility survey	clinic/hospital attendees / staff	≥ 40	316; 55		●			
Radwan [203]; 2014; Egypt	Self-designed	Cross-sectional worksite survey	Hospital workers	> 18	415; 81				●	
Rahhal [204]; 2017; Palestine	Self-designed	Cross-sectional worksite survey	Agricultural workers	15-76	98; Not available					●
Rahimi Rad [205]; 2007; Iran	ECRHS	Cross-sectional self-administered university survey	University students and staff	Mean age: 20.8 years	1,500; 95	●			●	
Rahimi Rad [206]; 2008; Iran	IUATLD	Cross-sectional interviewer-assisted house-to-house survey	General population	20-44	2,987; Not available	●				
Rahman [207]; 2013; Pakistan	Self-designed	Cross-sectional worksite survey	Brick kiln workers	25-65	814; Not available		●			
Ramirez-Venegas [208]; 2018; Mexico	PLATINO	Cross-sectional interviewer-assisted house-to-house survey	Women	> 35	1,333; Not available		●		●	
Rao [209]; 2011; India	Self-designed	Cross-sectional interviewer-assisted house-to-house survey	General population	No age limit	29,498; Not available	●				
Regalado [210]; 2006; Mexico	Self-designed	Cross-sectional self-administered house-to-house survey	Women	> 38	841; 97	●	●		●	●

Author; year; country	Questionnaire	Study description	Population	Age (in years)	Number of participants (n); response rate (%)	Measured outcomes				
						Asthma	COPD	Other CRDs	Symp toms	Lung function test
Rose [211]; 2006; Puerto Rico	National Health Interview Survey (NHIS)	Secondary data analysis	General population	> 18	95,615; Not available	●				
Rumana [212]; 2014; India	Health survey	Cross-sectional survey	General population	Not available	10,000; Not available	●	●			
Sakar [213]; 2006; Turkey	ECRHS	Cross-sectional interviewer-assisted house-to-house survey	General population	> 18	1,336; Not available	●				
Sallaoui [214]; 2007; Tunisia	USOC Sports Medicine Division questionnaire	Cross-sectional survey	Athletes	17-23	107; 100	●				
Sansores [215]; 2013; Mexico	Self-designed	Community (health fair) survey	Smokers	> 40	2,781; Not available		●			
Santos [216]; 2014; Brazil	ECRHS	Cross-sectional healthcare facility survey	Senior care centre users	> 50	318; Not available		●			
Saraclar [217]; 1997; Turkey	ECRHS	Community survey	General population	mean age: 34.5± 10	1,820; 90	●			●	
Schiavi [218]; 2014; Argentina, Colombia, Uruguay, Venezuela.	modified PLATINO	Cross-sectional healthcare facility survey	clinic/hospital attendees / staff	> 40	1,540; 88		●			
Sembajwe [219]; 2010; 64 countries	World Health Survey	Secondary data analysis	General population	> 18	308,218; Not available	●				
Shahzad [220]; 2006; Pakistan	Not reported	Cross-sectional worksite survey	Male leathery tannery workers	Mean age: 27 ± 9	641; Not available	●				

Author; year; country	Questionnaire	Study description	Population	Age (in years)	Number of participants (n); response rate (%)	Measured outcomes				
						Asthma	COPD	Other CRDs	Symp toms	Lung function test
Shaikh [221]; 2012; Pakistan	ATS-DLD-78-A	Cross-sectional worksite survey	Brick kiln workers	> 18	340; Not available	●		●	●	
Sharifi [222]; 2015; Iran	Previous Validated Questionnaire	Cross-sectional interviewer-assisted house-to-house survey	General population	18-40; >40	1,798; Not available		●		●	
Shishani [223]; 2006; Jordon	Respiratory Risk Profile (RRP)	Cross-sectional house-to-house survey	General population	> 18	400; Not available				●	
Sichletidis [224]; 2005; Greece	MRC questionnaire	Cross-sectional interviewer-assisted house-to-house survey	General population	21-80	6,112; 67 Thessaloniki, 77 Eordea, 87 Grevena		●			
Sichletidis [225]; 2011; Greece	IPAG	Cross-sectional healthcare facility survey	clinic/hospital attendees / staff	> 40	1,078; Not available		●			
Siddanagoudra [226]; 2014; India	Not available	Cross-sectional worksite survey	Spray paint workers	25-35	70; Not available	●				
Siddharthan [227]; 2018; 6 countries and 13 settings in Latin America, Sub-Saharan Africa, and Southeast Asia	Not available	Secondary data analysis	General population	35-95	12,396; Not available		●			
Sigari [228]; 2007; Iran	Not available	Cross-sectional worksite survey	Bakery workers	15-79	776; Not available	●				
Singh [229]; 2013; South Africa	ECRHS	Cross-sectional healthcare facility survey	Dental healthcare workers	Mean Age: 36	454; Not available	●				

Author; year; country	Questionnaire	Study description	Population	Age (in years)	Number of participants (n); response rate (%)	Measured outcomes				
						Asthma	COPD	Other CRDs	Symp toms	Lung function test
Sinha [230]; 2017; India	modified IPCAG questionnaire	Cross-sectional interviewer-assisted house-to-house survey	General population	> 30	1,203; 99		●			
Smeeton [231]; 2006; Chile	ECRHS	Cross-sectional survey	General population	22-28	601; Not available	●				
Smith [232]; 2014; China	CKB questionnaire	Cross-sectional healthcare facility survey	Never-smokers	30-79	317,000; Not available					●
Sobrino [233]; 2017; South America	self-designed, Global Adult Tobacco Survey	Cross-sectional interviewer-assisted healthcare facility survey	General population	45-74	4,354; 75		●			
Sonia [234]; 2018; Tunisia	ECRHS and ISAAC	Cross-sectional healthcare facility survey	General population	2-52	4,470; 97	●				
Sonomjamts [235]; 2014; Mongolia	ECRHS II and WHO questionnaire on major respiratory disease	Cross-sectional interviewer-assisted house-to-house survey	General population	> 20	1,201; Not available	●				
Soongkhang [236]; 2015; Thailand	ATS-DLD-78-A	Cross-sectional worksite survey	Wood manufacturing factory workers	18-59	511; Not available				●	
Stankovic [237]; 2011; Serbia	ATS	Cross-sectional interviewer-assisted healthcare facility survey	Women	20-40	1082; Not available	●				
Stephen [238]; 2018; India	INSEARCH	Cross-sectional interviewer-assisted worksite survey	Autorickshaw Drivers	> 21	297; Not available		●		●	
Sy [239]; 2007; Vietnam	ECRHS	Cross-sectional healthcare facility survey	General population	15-65	9,984; Not available	●			●	

Author; year; country	Questionnaire	Study description	Population	Age (in years)	Number of participants (n); response rate (%)	Measured outcomes				
						Asthma	COPD	Other CRDs	Symp toms	Lung function test
Tageldin [240]; 2012; Middle East & North Africa - Algeria, Egypt, Jordan, Lebanon, Morocco, Saudi Arabia, Syria, Tunisia, Turkey, UAE)	Not reported	Cross-sectional telephone survey	General population	> 40	62,086; Not available		●		●	
Tarraf [241]; 2018; Egypt, Turkey, Kuwait, Saudi Arabia, UAE	Global AIR studies screening questionnaire, Score for Allergic Rhinitis (SFAR) questionnaire and EQ-5D-3L questionnaire	Cross-sectional telephone survey	General population	> 18	33,486; 50.9	●				
Tiwari [242]; 1999; India	MRC questionnaire	Cross-sectional worksite survey	Handloom weavers	Not available	319; Not available					
To [243]; 2012; 70 countries	ISAAC, ECRHS	Secondary data analysis	General population	18-45	178,215; 98	●				
Toru [244]; 2014; Turkey	ATS	Cross-sectional worksite survey	Hairdressers, auto painters, carpenters	15-52	225; Not available				●	
Townend [245]; 2017; Tunisia, India, Sri Lanka, Nigeria, Morocco,	BOLD	Cross-sectional interviewer-assisted house-to-house survey	General population	> 40	9,255; 70					●

Author; year; country	Questionnaire	Study description	Population	Age (in years)	Number of participants (n); response rate (%)	Measured outcomes				
						Asthma	COPD	Other CRDs	Symp toms	Lung function test
Albania, Algeria, Malaysia, Saudi Arabia, Malawi, Kyrgyzstan										
Tug [246]; 2002; Turkey	ECRHS	Cross-sectional healthcare facility survey	General population	Mean Age: 37 years	3,591; Not available	●				
Tug [247]; 2002; Turkey	ECRHS	Cross-sectional healthcare facility survey	General population	Mean age:38.12 years ±12.17	2,454; 95	●			●	
Tzanakis [248]; 2004; Greece	Not available	Cross-sectional interviewer-assisted house-to-house survey	Smokers	> 35	888; Not available		●			
Ugurlu [249]; 2014; Turkey	ECRHS	Cross-sectional interviewer-assisted house-to-house survey	General population	> 18	1,343; Not available	●				
Uthaisangsook [250]; 2010; Thailand	ISAAC and modified ISAAC	Cross-sectional self-administered university survey	University students and staff	17-60	513; Not available	●				
Uthaisangsook [251]; 2007; Thailand	ISAAC	Cross-sectional self-administered university survey	University students and staff	17-53	2,693; Not available	●				
Valenti [252]; 2016; Brazil	Not mentioned	Cross-sectional interviewer-assisted house-to-house survey	General population	> 16	220; Not available					●
van Gemert [253]; 2015; Uganda	CCQ, mMRC questionnaire	Cross-sectional interviewer-assisted house-to-house survey	General population	> 30	588; 97		●			
Varona [254]; 2014; Philippines	ISAAC	Cross-sectional interviewer-assisted house-to-house survey	General population	> 20	7,202; 94	●				

Author; year; country	Questionnaire	Study description	Population	Age (in years)	Number of participants (n); response rate (%)	Measured outcomes				
						Asthma	COPD	Other CRDs	Symp toms	Lung function test
Vichyanond [255]; 2002; Thailand	ISAAC	Cross-sectional self-administered university survey	University students	16-31	3,631; >90	●				
Viinanen [256]; 2005; Mongolia	Tuohilampi Respiratory questionnaire	Cross-sectional interviewer-assisted house-to-house survey	General population	10 – 60	9,453; Not available	●				
Viswanathan [257]; 2018; India	IUATLD	Cross-sectional interviewer-assisted house-to-house survey	General population	> 15	12,556; Not available	●		●		
Waked [258]; 2011; Lebanon	ATS, MRC questionnaire	Cross-sectional healthcare facility survey	General population	> 40	2,201; 73		●			
Waked [259]; 2012; Lebanon	ATS, MRC questionnaire	Cross-sectional interviewer-assisted house-to-house survey	General population	> 40	2,201; 73		●	●		
Walraven [260]; 2001; Gambia	IUATLD	Cross-sectional healthcare facility survey	General population	> 15	2,166 (Banjul), 3,233 (Farafenni); 68 (Banjul) 87(Farafenni)	●			●	
Wang [261]; 2005; China	Self-designed	Prospective cohort study	Factory workers	Mean age: 56.3 years	559; 61			●	●	●
Wang [262]; 2013; China	National survey	Cross-sectional interviewer-assisted house-to-house survey	General population	All ages	13,419; 98	●				
Wang [263]; 2015; China	ATS-DLD-78-A	Cross-sectional survey	Farmers	> 40	728; 100				●	
Wang [264]; 2018; China	ECRHS	Cross-sectional interviewer-assisted house-to-house survey	General population	> 20	50,991; 93		●			

Author; year; country	Questionnaire	Study description	Population	Age (in years)	Number of participants (n); response rate (%)	Measured outcomes				
						Asthma	COPD	Other CRDs	Symp toms	Lung function test
Wilson [265]; 2008; China	ATS-DLD-78-A	Community survey	General population	Mean Age: 47.7years± 15.2	31,704; 83	●				
Xiao [266]; 2006; China	Not reported	Cross-sectional worksite survey	Factory workers	Mean age: 36.6	1,709; 94			●		
Xu [267]; 2001; China	ATS	Cross-sectional healthcare facility survey	General population	8 – 74	10,284; Not available	●			●	
Xu [268]; 2005; China	Not reported	Cross-sectional interviewer-assisted house-to-house survey	General population	> 35	29,319; 90		●			
Yamamoto [269]; 2000; Vietnam	ATS	Cross-sectional self-administered house-to-house survey	General population	30-49	1,416; Not available				●	
Yemaneberhan [270]; 1997; Ethiopia	IUATLD	Cross-sectional interviewer-assisted house-to-house survey	General population	All (0-120)	9,844 + 3,032; >95	●				
Yildiz [271]; 2010; Turkey	ECSC	Community survey	Men who spend long time in cafés	≥ 40	348; Not available		●			
Yin [272]; 2011; China	Self-designed	Cross-sectional interviewer-assisted house-to-house survey	General population	15-69	49,363; Not available		●			
Yingratanasuk [273]; 2002; Thailand	ATS	Cross-sectional worksite survey	Stone cavers	Not available	97; Not available				●	
Zhang [274]; 2002; China	IUATLD, ATS-DLD	Cross-sectional interviewer-assisted house-to-house survey	General population	> 15	22,528; 98	●			●	

Author; year; country	Questionnaire	Study description	Population	Age (in years)	Number of participants (n); response rate (%)	Measured outcomes				
						Asthma	COPD	Other CRDs	Symp toms	Lung function test
Zhang [275]; 2014; China	Recommended by the China Asthma Alliance	Cross-sectional interviewer-assisted house-to-house survey	General population	adult >14, children <14	19,861; Not available	●				
Zhang [276]; 2015; China	St. George's questionnaire	Cross-sectional survey	General population	> 6	27,042; Not available	●				
Zhong [277]; 2007; China	BOLD	Cross-sectional interviewer-assisted house-to-house survey	General population	> 40	20,245; 79		●			
Zhou [278]; 2009; China	BOLD	Cross-sectional house-to-house survey	Non-smokers	> 40	20,245; 79		●			
Zoller [279]; 2018; Tanzania	Self-designed	Cross-sectional healthcare facility survey	General population	> 18	598; Not available					●
Zubair [280]; 2017; Pakistan	Self-designed	Cross-sectional interviewer-assisted healthcare facility survey	Hospital attendees	18 - 60	517; 100		●			
Zuskin [281]; 2006; Croatia	European Union respiratory health questionnaire and WHO non-communicable diseases questionnaire	Cross-sectional interviewer-assisted house-to-house survey	General population	adults	1,001; Not available	●			●	

Notes and Abbreviation

ACQ - Asthma Control Questionnaire
ACT - Asthma Control Test
ATS - American Thoracic Society
ATS-DLD - American Thoracic Society Division of Lung Disease questionnaire
ATSDR - Agency for Toxic Substances and Disease Registry
BMRC - British Medical Research Council
BOLD - Burden of Obstructive Lung Disease
CAT - COPD Assessment Test
CCQ - Clinical COPD Questionnaire
CDC-BRFS Centers for Disease Control and Prevention Behavioral Risk Factor Surveillance System
CKB - China Kadoorie Biobank
ECRHS - European Community Respiratory Health Survey
ECSC - European Community for Coal and Steel
EQ-5D - EuroQol 5 Dimension
GA2LEN - Global Allergy and Asthma European Network
GARD - Global Alliance against Chronic Respiratory Diseases
INSEARCH- Indian study on epidemiology of asthma, respiratory symptoms and chronic bronchitis in adults
IPAG - International Primary Care Airways Guidelines
IPAQ - International Physical Activity Questionnaires
ISAAC - International Study of Asthma and Allergies in Childhood
IUALTD - International Union against Tuberculosis and Lung Disease (this was later revised and used in the ECRHS Study)
MRC questionnaire - Medical Research Council Respiratory Questionnaire
NHFS3 - National Family Health Survey 3
PLATINO - Latin American Project for the Investigation of Lung Disease
PUMA - used modified PLATINO
SF-12 - 12-Item Short Form Health Survey
USOC - United States Olympic Council
WHS - World Health Survey

REFERENCE

1 Abbasi IN, Ahsan A, Nafees AA. Correlation of respiratory symptoms and spirometric lung patterns in a rural community setting, Sindh, Pakistan: a cross sectional survey. BMC Pulm Med. 2012;12:81.

2 Abu Sham'a F, Skogstad M, Nijem K, Bjertness E, Kristensen P. Lung function and respiratory symptoms in male Palestinian farmers. Arch Environ Occup Health. 2010;65:191-200.

3 Aggarwal AN, Chaudhry K, Chhabra SK, Souza GA, Gupta D, Jindal SK, et al. Prevalence and risk factors for bronchial asthma in Indian adults: a multicentre study. Indian J Chest Dis Allied Sci. 2006;48:13-22.
4 Agrawal S. Effect of Indoor Air Pollution from Biomass and Solid Fuel Combustion on Prevalence of Self-Reported Asthma among Adult Men and Women in India: Findings from a Nationwide Large-Scale Cross-Sectional Survey. J Asthma. 2012;49:355-65.
5 Agrawal S, Pearce N, Ebrahim S. Prevalence and risk factors for self-reported asthma in an adult Indian population: a cross-sectional survey. Int J Tuberc Lung Dis. 2013;17:275-82.
6 Agrawal S, Pearce N, Millett C, Subramanian SV, Ebrahim S. Occupations with an increased prevalence of self-reported asthma in Indian adults. J Asthma. 2014;51:814-24.
7 Ahasan MR, Ahmad SA, Khan TP. Occupational exposure and respiratory illness symptoms among textile industry workers in a developing country. Appl Occup Environ Hyg. 2000;15:313-20.
8 Akkurt I, Sumer H, Ozsahin SL, Gonlugur U, Ozdemir L, Dogan O, et al. Prevalence of asthma and related symptoms in Sivas, Central Anatolia. Journal Asthma 2003;40:551-6.
9 Akpinar-Elci M, Cimrin AH, Elci OC. Prevalence and risk factors of occupational asthma among hairdressers in Turkey. J Occup Environ Med. 2002;44:585-90.
10 Akpinar-Elci M, Elci OC, Odabasi A. Work-related asthma-like symptoms among florists. Chest. 2004;125:2336-9.
11 Akpinar-Elci M, Coomansingh K, Blando J, Mark L. Household bush burning practice and related respiratory symptoms in Grenada, the Caribbean. Journal of the Air & Waste Management Association 2015;65:1148-52.
12 Akpinar-Elci M, Bidaisee S, Nguyen MT, Elci OC. Occupational exposure and respiratory health problems among nutmeg production workers in Grenada, the Caribbean. Int J Occup Environ Health. 2017;23:20-4.
13 Agarwal R, Dhooria S, Aggarwal AN, Maturu VN, Sehgal IS, Muthu V, et al. Guidelines for Diagnosis and Management of Bronchial Asthma: Joint Recommendations of National College of Chest Physicians (India) and Indian Chest Society. Indian J Chest Dis Allied Sci. 2015;57:5-52.
14 Amaral AFS, Patel J, Kato BS, Obaseki DO, Lawin H, Tan WC, et al. Airflow Obstruction and Use of Solid Fuels for Cooking or Heating BOLD (Burden of Obstructive Lung Disease) Results. Am J Respir Crit Care Med. 2018;197:595-610.
15 Amiri A, Asadi S, Almasi V, Ghobadi A. The Prevalence of Asthma in an Adult Population in Khorramabad, Iran. West Indian Medical Journal. 2014;63:443-6.
16 Andreeva E, Pokhaznikova M, Lebedev A, Moiseeva I, Kozlov A, Kuznetsova O, et al. The RESPECT study: RESearch on the PrEvalence and the diagnosis of COPD and its Tobacco-related etiology: a study protocol. BMC Public Health. 2015;15:831.
17 Andreeva E, Pokhaznikova M, Lebedev A, Moiseeva I, Kutznetsova O, Degryse JM. The Prevalence of Chronic Obstructive Pulmonary Disease by the Global Lung Initiative Equations in North-Western Russia. Respiration. 2016;91:43-55.
18 Arias SJ, Neffen H, Bossio JC, Calabrese CA, Videla AJ, Armando GA, et al. Prevalence and Features of Asthma in Young Adults in Urban Areas of Argentina. Arch Bronconeumol. 2018;54:134-9.
19 Arora S, Rasania SK, Bachani D, Gandhi A, Chhabra SK. Air pollution and environmental risk factors for altered lung function among adult women of an urban slum area of Delhi: A prevalence study. Lung India. 2018;35:193-8.

20 Arslan Z, Ilgazli A, Etiler N, Hamzaoglu O. Prevalence of Chronic Obstructive Pulmonary Disease in Kocaeli: An Industrialised City in Turkey. Balkan Med J. 2013;30:387-93.
21 Athavale A, Iyer H, Punwani AD, Shah JJ, Natraj G, Nair JP, et al. Association of Environmental Factors, Prevalence of Asthma and Respiratory Morbidity in Mumbai: Need of a Public Health Policy. The Journal of the Association of Physicians of India. 2017;65:48-54.
22 Baatjies R, Lopata AL, Sander I, Raulf-Heimsoth M, Bateman ED, Meijster T, et al. Determinants of asthma phenotypes in supermarket bakery workers. Eur Respir J. 2009;34:825-33.
23 Badway MS, Hamed AF, Yousef FMA. Prevalence of chronic obstructive pulmonary disease (COPD) in Qena Governorate. Egypt J Chest Dis Tuberc. 2016;65:29-34.
24 Balcan B, Akan S, Ozsancak Ugurlu A, Ceyhan B. Altered pulmonary functions due to biomass smoke in a rural population of Turkish women: A descriptive study. Tuberk Toraks. 2018;66:122-9.
25 Banda HT, Thomson R, Mortimer K, Bello GAF, Mbera GB, Malmborg R, et al. Community prevalence of chronic respiratory symptoms in rural Malawi: Implications for policy. PloS one. 2017;12:e0188437.
26 Barbieri MA, Bettiol H, Silva AAM, Cardoso VC, Simoes VMF, Gutierrez MRP, et al. Health in early adulthood: the contribution of the 1978/79 Ribeirao Preto birth cohort. Brazilian journal of medical and biological research = Revista brasileira de pesquisas medicas e biologicas. 2006;39:1041-55.
27 Baris SA, Yildiz F, Basyigit I, Boyaci H, Ilgazli A. Prevalence of smoking and chronic obstructive pulmonary disease amongst teachers working in Kocaeli, Turkey. Multidiscip Respir Med. 2011;6:92-6.
28 Boskabady MH, Kolahdoz GH. Prevalence of asthma symptoms among the adult population in the city of Mashhad (north-east of Iran). Respirology (Carlton, Vic). 2002;7:267-72.
29 Budhathoki SS, Singh SB, Niraula SR, Pokharel PK. Morbidity patterns among the welders of eastern Nepal: A cross-sectional study. Ann Occup Environ Med. 2016;28:62.
30 Buist AS, Vollmer WM, McBurnie MA. Worldwide burden of COPD in high- and low-income countries. Part I. The burden of obstructive lung disease (BOLD) initiative. Int J Tuberc Lung Dis. 2008;12:703-8.
31 Caballero A, Torres-Duque CA, Jaramillo C, Bolivar F, Sanabria F, Osorio P, et al. Prevalence of COPD in five Colombian cities situated at low, medium, and high altitude (PREPOCOL study). Chest. 2008;133:343-9.
32 Caban-Martinez AJ, Halder GE, Tellechea L, Fajardo M, Kaltman J, Anand J, et al. Health status and behaviors among adults residing in rural Dominican Republic. Rural Remote Health. 2012;12.
33 Caldeira RD, Bettiol H, Barbieri MA, Terra J, Garcia CA, Vianna EO. Prevalence and risk factors for work related asthma in young adults. Occup Environ Med. 2006;63:694-9.
34 Cardoso LS, Costa DM, Almeida MCF, Souza RP, Carvalho EM, Araujo MI, et al. Risk factors for asthma in a Helminth endemic area in Bahia, Brazil. J Parasitol Res. 2012.
35 Celedon JC, Silverman EK, Weiss ST, Wang B, Fang Z, Xu X. Application of an algorithm for the diagnosis of asthma in Chinese families: Limitations and alternatives for the phenotypic assessment of asthma in family-based genetic studies. Am J Respir Crit Care Med. 2000;162:1679-84.

36 Celik G, Mungan D, Bavbek S, Sin B, Ediger D, Demirel Y, et al. The prevalence of allergic diseases and atopy in Ankara, Turkey: a two-step population-based epidemiological study. J Asthma. 1999;36:281-90.

37 Chan YY, Teh CH, Lim KK, Lim KH, Yeo PS, Kee CC, et al. Lifestyle, chronic diseases and self-rated health among Malaysian adults: results from the 2011 National Health and Morbidity Survey (NHMS). BMC Public Health. 2015;15:754.

38 Chan-Yeung M, Zhang LX, Tu DH, Li B, He GX, Kauppinen R, et al. The prevalence of asthma and asthma-like symptoms among adults in rural Beijing, China. Eur Respir J. 2002;19:853-8.

39 Chattopadhyay K, Chattopadhyay C, Kaltenthaler E. Respiratory health status and its predictors: a cross-sectional study among coal-based sponge iron plant workers in Barjora, India. BMJ Open. 2015;5.

40 Chhabra SK, Chhabra P, Rajpal S, Gupta RK. Ambient air pollution and chronic respiratory morbidity in Delhi. Arch Environ Health. 2001;56:58-64.

41 Chien VC, Chai SK, Hai DN, Takaro T, Checkoway H, Keifer M, et al. Pneumoconiosis among workers in a Vietnamese refractory brick facility. Am J Ind Med. 2002;42:397-402.

42 Ching S-M, Pang Y-K, Price D, Cheong A-T, Lee P-Y, Irmi I, et al. Detection of airflow limitation using a handheld spirometer in a primary care setting. Respirology (Carlton, Vic). 2014;19:689-93.

43 Chkhaidze I, Maglakelidze T, Khaltaev N. Chronic respiratory diseases at primary health care level in Georgia: The results of the pilot study. Monaldi Archives for Chest Disease - Pulmonary Series. 2009;71:141-6.

44 Chowgule RV, Shetye VM, Parmar JR, Bhosale AM, Khandagale MR, Phalnitkar SV, et al. Prevalence of respiratory symptoms, bronchial hyperreactivity, and asthma in a megacity. Results of the European community respiratory health survey in Mumbai (Bombay). Am J Respir Crit Care Med. 1998;158:547-54.

45 Choy DKL, Hui DSC, Li ST, Ko FWS, Ho S, Woo J, et al. Prevalence of wheeze, bronchial hyper-responsiveness and asthma in the elderly Chinese. Clin Exp Allergy. 2002;32:702-7.

46 Chuaychoo B, Naruman C, Lertakyamanee J, Nana A, Suthamsmai T, Sreelum W, et al. The Most Cost-Effective Screening Method for Chronic Obstructive Pulmonary Disease among the Bangkok Elderly. J Med Assoc Thai. 2003;86:1140-8.

47 Chuchalin AG, Khaltaev N, Antonov NS, Galkin DV, Manakov LG, Antonini P, et al. Chronic respiratory diseases and risk factors in 12 regions of the Russian Federation. Int J Chron Obstruct Pulmon Dis. 2014;9:963-74.

48 Daldoul H, Denguezli M, Jithoo A, Gnatiuc L, Buist S, Burney P, et al. Prevalence of COPD and Tobacco Smoking in Tunisia - Results from the BOLD Study. International Journal of Environmental Research and Public Health. 2013;10:7257-71.

49 Davey G, Venn A, Belete H, Berhane Y, Britton J. Wheeze, allergic sensitization and geohelminth infection in Butajira, Ethiopia. Clin Exp Allergy. 2005;35:301-7.

50 de Fatima Macaira E, Algranti E, Medina Coeli Mendonca E, Antonio Bussacos M. Rhinitis and asthma symptoms in non-domestic cleaners from the Sao Paulo metropolitan area, Brazil. Occup Environ Med. 2007;64:446-53.

51 de Oca MM, Halbert RJ, Lopez MV, Perez-Padilla R, Tálamo C, Moreno D, et al. The chronic bronchitis phenotype in subjects with and without COPD: the PLATINO study. Eur Respir J. 2012;40:28-36.

52 Montes de Oca M, Victorina Lopez Varela M, Laucho-Contreras ME, Casas A, Schiavi E, Mora JC. Asthma-COPD overlap syndrome (ACOS) in primary care of four Latin America countries: The PUMA study. BMC Pulm Med. 2017;17:69.
53 de Sousa CA, Cesar CLG, Barros MBD, Carandina L, Goldbaum M, Pereira JCR. Prevalence of chronic obstructive pulmonary disease and risk factors in Sao Paulo, Brazil, 2008-2009. Rev Saude Publica. 2011;45:887-96.
54 de Souza RM, de Andrade FM, Moura ABD, Teixeira PJZ. Respiratory symptoms in charcoal production workers in the cities of Lindolfo Collor, Ivoti and Presidente Lucena, Brazil. J Bras Pneumol. 2010;36:210-7.
55 Dejsomritrutai W, Nana A, Chierakul N, Tscheikuna J, Sompradeekul S, Ruttanaumpawan P, et al. Prevalence of bronchial hyperresponsiveness and asthma in the adult population in Thailand. Chest. 2006;129:602-9.
56 Dejsomritrutai W, Siritantikorn S, Nana A. Asthma, bronchial hyper-responsiveness and Chlamydophila (Chlamydia) pneumonia infection in adult Thai population. J Med Assoc Thai. 2009;92 Suppl 2:S30-7.
57 Denguezli M, Daldoul H, Harrabi I, Gnatiuc L, Coton S, Burney P, et al. COPD in Nonsmokers: Reports from the Tunisian Population-Based Burden of Obstructive Lung Disease Study. PloS one. 2016;11:e0151981.
58 Dennis RJ, Caraballo L, Garcia E, Rojas MX, Rondon MA, Perez A, et al. Prevalence of asthma and other allergic conditions in Colombia 2009-2010: a cross-sectional study. BMC Pulm Med. 2012;12:17.
59 Desalu OO, Salami AK, Oluboyo PO. Self-reported risk factors of asthma in a nigerian adult population. Turk Thorac J. 2009;10:56-62.
60 Desalu OO. Prevalence of chronic bronchitis and tobacco smoking in some rural communities in Ekiti state, Nigeria. Niger Postgrad Med J. 2011;18:91-7.
61 Deschamps F, Sow ML, Prevost A, Henry L, Lavaud F, Bernard J, et al. Prevalence of respiratory symptoms and increased specific IgE levels in West-African workers exposed to isocyanates. Journal of toxicology and environmental health Part A. 1998;54:335-42.
62 Deveci F, Deveci SE, Turkoglu S, Turgut T, Kirkil G, Rahman S, et al. The prevalence of chronic obstructive pulmonary disease in Elazig, Eastern Turkey. Eur J Intern Med. 2011;22:172-6.
63 Diaz E, Bruce N, Pope D, Lie RT, Diaz A, Arana B, et al. Lung function and symptoms among indigenous Mayan women exposed to high levels of indoor air pollution. Int J Tuberc Lung Dis. 2007;11:1372-9.
64 Ding YP, Yao HX, Tang XL, He HW, Shi HF, Lin L, et al. An epidemiology study of bronchial asthma in the Li ethnic group in. China. Asia Pac J Public Health. 2012;5:157-61.
65 Ding YP, Xu JX, Yao JJ, Chen Y, He P, Ouyang YH, et al. The analyses of risk factors for COPD in the Li ethnic group in Hainan, People's Republic of China. Int J Chron Obstruct Pulmon Dis. 2015;10:2593-9.
66 Ding Y, Yang D, He P, Yao J, Sun P, Li Q, et al. Prevalence and risk factors of chronic obstructive pulmonary diseases in a Hlai community in Hainan Island of China. Clin Respir J. 2018;12:126-33.
67 Dong GH, Qian Z, Wang J, Trevathan E, Ma W, Chen W, et al. Residential characteristics and household risk factors and respiratory diseases in Chinese women: The Seven Northeast Cities (SNEC) Study. Sci Total Environ. 2013;463-464:389-94.
68 Dutta S, Deshmukh PR. Prevalence and determinants of self-reported chronic bronchitis among women in rural Central India. Med J Armed Forces India. 2015;71:48-52.

69 Echazarreta AL, Arias SJ, del Olmo R, Giugno ER, Colodenco FD, Arce SC, et al. Prevalence of COPD in 6 Urban Clusters in Argentina: The EPOC.AR Study. Arch Bronconeumol. 2018;54:260-9.

70 Ehrlich RI, White N, Norman R, Laubscher R, Steyn K, Lombard C, et al. Predictors of chronic bronchitis in South African adults. Int J Tuberc Lung Dis. 2004;8:369-76.

71 El Hasnaoui A, Rashid N, Lahlou A, Salhi H, Doble A, Nejjari C, et al. Chronic obstructive pulmonary disease in the adult population within the Middle East and North Africa region: rationale and design of the BREATHE study. Respir Med. 2012;106:S3-S15.

72 El Rhazi K, Nejjari C, Ben Jelloun MC, El Biaze M, Attassi M, Garcia-Larsen V. Prevalence of chronic obstructive pulmonary disease in Fez, Morocco: Results from the BOLD study. Int J Tuberc Lung Dis. 2016;20:136-41.

73 Erhabor GE, Agbroko SO, Bamigboye P, Awopeju OF. Prevalence of asthma symptoms among university students 15 to 35 years of age in Obafemi Awolowo University, Ile-Ife, Osun State. The Journal of asthma : official journal of the Association for the Care of Asthma. 2006;43:161-4.

74 Erhabor GE, Obaseki DO, Awopeju OF, Ijadunola KT, Adewole OO. Asthma in a university campus: a survey of students and staff of Obafemi Awolowo University, Ile-Ife, Nigeria. J Asthma. 2016;53:30-6.

75 Fazlollahi MR, Najmi M, Fallahnezhad M, Sabetkish N, Kazemnejad A, Bidad K, et al. The prevalence of asthma in Iranian adults: The first national survey and the most recent updates. Clin Respir J. 2018;12:1872-81.

76 Fereidouni M, Abolhasani A, Vahedi F, Shakeri MT, Varasteh A. A preliminary survey of the prevalence of allergic disorders in a questionnaire-based study in Boshroye, a rural area of Iran. J Public Health. 2010;18:119-21.

77 Franco-Marina F, Fernandez-Plata R, Torre-Bouscoulet L, Garcia-Sancho C, Sanchez-Gallen E, Martinez D, et al. Efficient screening for COPD using three steps: a cross-sectional study in Mexico City. NPJ Prim Care Respir Med. 2014;24:1-8.

78 Gathuru IM, Bunker CH, Ukoli FA, Egbagbe EE. Differences in rates of obstructive lung disease between Africans and African Americans. Ethnicity and Disease. 2002;12:S3-113.

79 Ghasemkhani M, Kumashiro M, Rezaei M, Anvari AR, Mazloumi A, Sadeghipour HR. Prevalence of respiratory symptoms among workers in industries of south Tehran, Iran. Ind Health. 2006;44:218-24.

80 Gizaw Z, Yifred B, Tadesse T. Chronic respiratory symptoms and associated factors among cement factory workers in Dejen town, Amhara regional state, Ethiopia, 2015. Multidiscip Respir Med. 2016;11:13.

81 Golshan M, Esteki B, Dadvand P. Prevalence of self-reported respiratory symptoms in rural areas of Iran in 2000. Respirology (Carlton, Vic). 2002;7:129-32.

82 Golshan M, Barahimi H, Nasirian K. Prevalence of chronic bronchitis and chronic respiratory symptoms in adults over the age of 35 years in Isfahan, Iran in 1998. Respirology (Carlton, Vic). 2001;6:231-5.

83 Golshan M, Faghihi M, Roushan-Zamir T, Marandi MM, Esteki B, Dadvand P, et al. Early effects of burning rice farm residues on respiratory symptoms of villagers in suburbs of Isfahan, Iran. Int J Environ Health Res. 2002;12:125-31.

84 Golshan M, Amra B, Welte T. Sample survey of chronic obstructive pulmonary disease and associated risk factors in Isfahan, Iran. Tanaffos. 2011;10:32-6.

85 Gonzalez-Garcia M, Caballero A, Jaramillo C, Maldonado D, Torres-Duque CA. Prevalence, risk factors and underdiagnosis of asthma and wheezing in adults 40 years and older: A population-based study. J Asthma. 2015;52:823-30.
86 Gorgieva GS, Stasevic Z, Vasic S, Ristic S, Lezaic V, Djukanovic L. Screening of chronic diseases and chronic disease risk factors in two rural communities in Kosovo. Cent Eur J Public Health. 2010;18:81-6.
87 Gourgoulianis KI, Katikos P, Moraitis M, Argiriou N, Molyvdas PA. Chronic bronchitis in rural and industrial areas. Ann Agric Environ Med. 2000;7:29-31.
88 Guddattu V, Swathi A, Sreekumaran N. Household and Environment Factors Associated With Asthma Among Indian Women: A Multilevel Approach. J Asthma. 2010;47:407-11.
89 Gunen H, Hacievliyagil SS, Yetkin O, Gulbas G, Mutlu LC, Pehlivan E. Prevalence of COPD: first epidemiological study of a large region in Turkey. Eur J Intern Med. 2008;19:499-504.
90 Gupta D, Aggarwal AN, Chaudhry K, Chhabra SK, D'Souza GA, Jindal SK, et al. Household environmental tobacco smoke exposure, respiratory symptoms and asthma in non-smoker adults: a multicentric population study from India. Indian J Chest Dis Allied Sci. 2006;48:31-6.
91 Gupta S, Arora V, Sharma OP, Satyanarayana L, Gupta AK. Prevalence & pattern of respiratory diseases including Tuberculosis in elderly in Ghaziabad - Delhi - NCR. Indian J Tuberc. 2016;63:236-41.
92 Gupta BG, Biswas JK, Agrawal KM. Air Pollution From Bleaching and Dyeing Industries Creating Severe Health Hazards in Maheshtala Textile Cluster, West Bengal, India. Air Soil and Water Research. 2017;10:1-10.
93 Hamatui N, Beynon C. Particulate Matter and Respiratory Symptoms among Adults Living in Windhoek, Namibia: A Cross Sectional Descriptive Study. International Journal of Environmental Research and Public Health 2017;14.
94 Hamzaçebi H, Unsal M, Kayhan S, Bilgin S, Ercan S. Prevalence of asthma and respiratory symptoms by age, gender and smoking behaviour in Samsun, North Anatolia Turkey. Tuberk Toraks. 2006;54:322-9.
95 Han YY, Forno E, Canino G, Celedon JC. Psychosocial risk factors and asthma among adults in Puerto Rico. J Asthma. 2018:1-9.
96 Horner A, Soriano JB, Puhan MA, Studnicka M, Kaiser B, Vanfleteren LEGW, et al. Altitude and COPD prevalence: analysis of the PREPOCOL-PLATINO-BOLD-EPI-SCAN study. Respir Res. 2017;18:162.
97 Huang X, Chen MS, Tan HZ, Xiao SY, Deng J. The Morbidity Rate of Chronic Disease among Chinese Rural Residents: Results from Liuyang Cohort. Medical Principles and Practice. 2013;22:362-7.
98 Idolor LF, De Guia TS, Francisco NA, Roa CC, Ayuyao FG, Tady CZ, et al. Burden of obstructive lung disease in a rural setting in the Philippines. Respirology. 2011;16:1111-8.
99 Ishtiaq M, Rabnawaz, Khan K, Khan H, Zakir S, Sarwar G, et al. Prevalance of pneumoconiosis among coal miners of Cherat, District Nowshera - Pakistan. J Postgrad Med Inst. 2014;28:139-44.
100 Jaganath D, Miranda JJ, Gilman RH, Wise RA, Diette GB, Miele CH, et al. Prevalence of chronic obstructive pulmonary disease and variation in risk factors across four geographically diverse resource-limited settings in Peru. Respir Res. 2015;16:40.
101 Jeebhay MF, Robins TG, Miller ME, Bateman E, Smuts M, Baatjies R, et al. Occupational Allergy and Asthma Among Salt Water Fish Processing Workers. Am J Ind Med. 2008;51:899-910.

102 Jie Y, Isa ZM, Jie X, Ismail NH. Asthma and Asthma-Related Symptoms among Adults of an Acid Rain-Plagued City in Southwest China: Prevalence and Risk Factors. Pol J Environ Stud. 2013;22:717-26.

103 Jie Y, Kebin L, Yin T, Jie X. Indoor Environmental Factors and Occurrence of Lung Function Decline in Adult Residents in Summer in Southwest China. Iran J Public Health. 2016;45:1436-45.

104 Jie Y, Li KB, Yin T, Jie X. Prevalence of Asthma and Asthma-Related Symptoms Among Adults Exposed to Indoor Environmental Risk Factors: a Comparison between Winter and Summer in Zunyi, China. Pol J Environ Stud. 2016;25:621-33.

105 Jindal SK, Gupta D, Aggarwal AN, Jindal RC, Singh V. Study of the prevalence of asthma in adults in North India using a standardized field questionnaire. J Asthma. 2000;37:345-51.

106 Jindal SK, Aggarwal AN, Chaudhry K, Chhabra SK, Souza GA, Gupta D, et al. A multicentric study on epidemiology of chronic obstructive pulmonary disease and its relationship with tobacco smoking and environmental tobacco smoke exposure. Indian J Chest Dis Allied Sci. 2006;48:23-9.

107 Jindal SK, Aggarwal AN, Gupta D, Agarwal R, Kumar R, Kaur T, et al. Indian Study on Epidemiology of Asthma, Respiratory Symptoms and Chronic Bronchitis in adults (INSEARCH). Int J Tuberc Lung Dis. 2012;16:1270-7.

108 Johnson P, Balakrishnan K, Ramaswamy P, Ghosh S, Sadhasivam M, Abirami O, et al. Prevalence of chronic obstructive pulmonary disease in rural women of Tamilnadu: implications for refining disease burden assessments attributable to household biomass combustion. Glob Health Action. 2011;4:7226-.

109 Kahwa EK, Younger NO, Wint YB, Waldron NK, Hewitt HH, Knight-Madden JM, et al. The Jamaica asthma and allergies national prevalence survey: rationale and methods. BMC Med Res Methodol. 2010;10:29.

110 Kavishe B, Biraro S, Baisley K, Vanobberghen F, Kapiga S, Munderi P, et al. High prevalence of hypertension and of risk factors for non-communicable diseases (NCDs): A population based cross-sectional survey of NCDS and HIV infection in Northwestern Tanzania and Southern Uganda. BMC Med. 2015;13:126.

111 Ko FWS, Woo J, Tam W, Lai CKW, Ngai J, Kwok T, et al. Prevalence and risk factors of airflow obstruction in an elderly Chinese population. Eur Respir J. 2008;32:1472-8.

112 Konuk S, Tug T. The prevalence of chronic obstructive pulmonary disease in Bolu province of Turkey. Journal of Clinical and Analytical Medicine. 2017;8:346-9.

113 Konuk S, Coban H. COPD Prevalence in Sirnak City Center. Journal of Clinical and Analytical Medicine. 2017;8:379-82.

114 Koul PA, Hakim NA, Malik SA, Khan UH, Patel J, Gnatiuc L, et al. Prevalence of chronic airflow limitation in Kashmir, North India: results from the BOLD study. Int J Tuberc Lung Dis. 2016;20:1399-404.

115 Kourlaba G, Bakakos P, Loukides S, Vellopoulou K, Solakidi A, Maniadakis N. The self-reported prevalence and disease burden of asthma in Greece. J Asthma. 2018:1-20.

116 Prashanth Kumar SP, Parasuramalu BG, Huliraj N, Gangaboraiah, Ramesh Masthi NR, Srinivasa Babu CR. Assessing the burden of bronchial asthma in rural adult population of Bangalore. Indian J Public Health Res Dev. 2017;8:59-64.

117 Kurmi OP, Devereux GS, Smith WCS, Semple S, Steiner MFC, Simkhada P, et al. Reduced lung function due to biomass smoke exposure in young adults in rural Nepal. Eur Respir J. 2013;41:25-30.

118 Kurmi OP, Millwood IY, Li J, Chen B, Xie K, On behalf of the China Kadoorie Biobank Collaborative G, et al. COPD and its association with smoking in the Mainland China: A cross-sectional analysis of 0.5 million men and women from ten diverse areas. Int J Chron Obstruct Pulmon Dis. 2015;10:655-65.
119 Kurt E, Demir AU, Cadirci O, Yildirim H, Ak G, Eser TP. Occupational exposures as risk factors for asthma and allergic diseases in a Turkish population. International Archive of Occupational and Environmental Health. 2011;84:45-52.
120 Lai CK, Ho SC, Lau J, Yuen YK, Ho SS, Chan CH, et al. Respiratory symptoms in elderly Chinese living in Hong Kong. Eur Respir J. 1995;8:2055-61.
121 Lai K, Chen R, Lin J, Huang K, Shen H, Kong L, et al. A prospective, multicenter survey on causes of chronic cough in China. Chest. 2013;143:613-20.
122 Lam KBH, Jiang CQ, Jordan RE, Miller MR, Zhang WS, Cheng KK, et al. Prior TB, Smoking, and Airflow Obstruction A Cross-Sectional Analysis of the Guangzhou Biobank Cohort Study. Chest. 2010;137:593-600.
123 Hoang TL, Ronmark E, Nguyen VT, Ekerljung L, Nguyen TKC, Lundback B. Increase in asthma and a high prevalence of bronchitis: Results from a population study among adults in urban and rural Vietnam. Respir Med. 2011;105:177-85.
124 Lam KBH, Yin P, Jiang CQ, Sen Zhang W, Adab P, Miller MR, et al. Past dust and GAS/FUME exposure and COPD in Chinese: The Guangzhou Biobank Cohort Study. Respir Med. 2012;106:1421-8.
125 Lam HT, Ekerljung L, Nguy TN, Ronmark E, Larsson K, Lundback B. Prevalence of COPD by disease severity in men and women in Northern Vietnam. COPD. 2014;11:575-81.
126 Lamprecht B, McBurnie MA, Vollmer WM, Gudmundsson G, Welte T, Nizankowska-Mogilnicka E, et al. COPD in never smokers: Results from the population-based burden of obstructive lung disease study. Chest. 2011;139:752-63.
127 Laniado-Laborin R, Rendon A, Bauerle O. Chronic obstructive pulmonary disease case finding in Mexico in an at-risk population. Int J Tuberc Lung Dis. 2011;15.
128 Laraqui O, Hammouda R, Laraqui S, Manar N, Ghailan T, Ben Amor J, et al. Prevalence of chronic obstructive respiratory diseases amongst fishermen. Intern Marit Health. 2018;69:13-21.
129 Lim HH, Rampal KG, Joginder S, Abu Bakar CM, Chan KH, Vivek TN. Respiratory conditions in Malaysian asbestos cement workers. Med J Malaysia. 2002;57:340-7.
130 Lin S, Lawrence WR, Lin Z, Francois M, Neamtiu IA, Lin Q, et al. Teacher respiratory health symptoms in relation to school and home environment. International archives of occupational and environmental health. 2017;90:725-39.
131 Lin JT, Wang WY, Chen P, Zhou X, Wan HY, Yin KS, et al. Prevalence and risk factors of asthma in mainland China: The CARE study. Respir Med. 2018;137:48-54.
132 Liu SM, Zhou YM, Wang XP, Wang DL, Lu JC, Zheng JP, et al. Biomass fuels are the probable risk factor for chronic obstructive pulmonary disease in rural South China. Thorax. 2007;62:889-97.
133 Liu S, Ren Y, Wen D, Chen Y, Chen D, Li L, et al. Prevalence and risk factors for COPD in greenhouse farmers: a large, cross-sectional survey of 5,880 farmers from northeast China. Int J Chron Obstruct Pulmon Dis. 2015;10:2097-108.
134 Liu S, Zhou Y, Liu S, Chen X, Zou W, Zhao D, et al. Association between exposure to ambient particulate matter and chronic obstructive pulmonary disease: results from a cross-sectional study in China. Thorax. 2017;72:788-95.

135 Loh LC, Rashid A, Sholehah S, Gnatiuc L, Patel JH, Burney P. Low prevalence of obstructive lung disease in a suburban population of Malaysia: A BOLD collaborative study. Respirology. 2016;21:1055-61.

136 Lopez Varela MV, Montes de Oca M, Halbert R, Muino A, Talamo C, Perez-Padilla R, et al. Comorbidities and health status in individuals with and without COPD in five Latin American cities: the PLATINO study. Arch Bronconeumol. 2013;49:468-74.

137 Lopez Varela MV, Montes de Oca M, Rey A, Casas A, Stirbulov R, Di Boscio V, et al. Development of a simple screening tool for opportunistic COPD case finding in primary care in Latin America: The PUMA study. Respirology. 2016;21:1227-34.

138 Lu M, Yao W-z, Zhong N-s, Zhou Y-m, Wang C, Chen P, et al. Asymptomatic patients of chronic obstructive pulmonary disease in China. Chin Med J. 2010;123:1494-9.

139 Luenam A, Laohasiriwong W, Puttanapong N, Saengsuwan J, Phajan T. Socioeconomic disparities and chronic respiratory diseases in Thailand: The National Socioeconomics Survey. Inform Health Soc Care. 2018;43:348-61.

140 Mackenney J, Oyarzun MJ, Diaz RV, Bustos R, Amigo H, Rona RJ. Prevalence of asthma, atopy and bronchial hyperresponsiveness and their interrelation in a semi-rural area of Chile. Int J Tuberc Lung Dis. 2005;9:1288-93.

141 Magitta NF, Walker RW, Apte KK, Shimwela MD, Mwaiselage JD, Sanga AA, et al. Prevalence, risk factors and clinical correlates of COPD in a rural setting in Tanzania. Eur Respir J. 2018;51.

142 Mahesh P, Jayaraj B, Prahlad S, Chaya S, Prabhakar A, Agarwal A, et al. Validation of a structured questionnaire for COPD and prevalence of COPD in rural area of Mysore: A pilot study. Lung India. 2009;26:63-9.

143 Mahesh PA, Jayaraj BS, Prabhakar AK, Chaya SK, Vijayasimha R. Prevalence of chronic cough, chronic phlegm & associated factors in Mysore, Karnataka, India. Ind J Med Res. 2011;134:91-100.

144 Mahesh PA, Jayaraj BS, Chaya SK, Lokesh KS, McKay AJ, Prabhakar AK, et al. Variation in the prevalence of chronic bronchitis among smokers: A cross-sectional study. Int J Tuberc Lung Dis. 2014;18:862-9.

145 Mamane A, Tessier JF, Bouvier G, Salamon R, Lebailly P, Raherison C, et al. Increase in the Risk of Respiratory Disorders in Adults and Children Related to Crop-Growing in Niger. J Environ Public Health. 2016.

146 Mannino DM, McBurnie MA, Tan W, Kocabas A, Anto J, Vollmer WM, et al. Restricted spirometry in the Burden of Lung Disease Study. Int J Tuberc Lung Dis. 2012;16:1405-11.

147 Maranetra KN, Chuaychoo B, Dejsomritrutai W, Chierakul N, Nana A, Lertakyamanee J, et al. The prevalence and incidence of COPD among urban older persons of Bangkok Metropolis. J Med Assoc Thai. 2002;85:1147-55.

148 Maranetra N, Lertakyamanee J, Chierakul N, Nana A, Suthamsmai T, Sreelum W, et al. The Cost-Effectiveness of a Questionnaire as a Screening Test for Chronic Obstructive Pulmonary Disease among the Bangkok Elderly. J Med Assoc Thai. 2003;86:1033-41.

149 Masoompour SM, Mahdaviazad H, Ghayumi SMA. Asthma and its related socioeconomic factors: The Shiraz Adult Respiratory Disease Study 2015. Clin Respir J. 2018;12:2110-6.

150 Mberikunashe J, Banda S, Chadambuka A, Gombe NT, Shambira G, Tshimanga M, et al. Prevalence and risk factors for obstructive respiratory conditions among textile industry workers in Zimbabwe, 2006. Pan Afr Med J. 2010;6.

151 Meghji J, Nadeau G, Davis KJ, Wang DL, Nyirenda MJ, Gordon SB, et al. Noncommunicable Lung Disease in Sub-Saharan Africa A Community-based Cross-Sectional Study of Adults in Urban Malawi. Am J Respir Crit Care Med. 2016;194:67-76.
152 Mejza F, Gnatiuc L, Buist AS, Vollmer WM, Lamprecht B, Obaseki DO, et al. Prevalence and burden of chronic bronchitis symptoms: results from the BOLD study. Eur Respir J. 2017;50.
153 Meneghini AC, Paulino ACB, Pereira LP, Vianna EO. Accuracy of spirometry for detection of asthma: a cross-sectional study. Sao Paulo Med J. 2017;135:428-33.
154 Menezes A, Macedo SC, Gigante DP, da Costa JD, Olinto MT, Fiss E, et al. Prevalence and risk factors for chronic obstructive pulmonary disease according to symptoms and spirometry. COPD. 2004;1:173-9.
155 Menezes AM, Jardim JR, Perez-Padilla R, Camelier A, Rosa F, Nascimento O, et al. Prevalence of chronic obstructive pulmonary disease and associated factors: the PLATINO Study in Sao Paulo, Brazil. Cadernos de saude publica / Ministerio da Saude, Fundacao Oswaldo Cruz, Escola Nacional de Saude Publica. 2005;21:1565-73.
156 Menezes AMB, Perez-Padilla R, Jardim JRB, Muino A, Lopez MV, Valdivia G, et al. Chronic obstructive pulmonary disease in five Latin American cities (the PLATINO study): a prevalence study. Lancet 2005;366:1875-81.
157 Menezes AM, Lima RC, Minten GC, Hallal PC, Victora CG, Horta BL, et al. Prevalence of wheezing in the chest among adults from the 1982 Pelotas birth cohort, Southern Brazil. Rev Saude Publica. 2008;42 Suppl 2:101-7.
158 Menezes AMB, Perez-Padilla R, Hallal PC, Jardim JR, Muino A, Lopez MV, et al. Worldwide burden of COPD in high- and low-income countries. Part II. Burden of chronic obstructive lung disease in Latin America: the PLATINO study. Int J Tuberc Lung Dis. 2008;12:709-12.
159 Menezes AM, Wehrmeister FC, Horta B, Szwarcwald CL, Vieira ML, Malta DC. Prevalence of asthma medical diagnosis among Brazilian adults: National Health Survey, 2013. Rev Bras Epidemiol. 2015;18:204-13.
160 Menezes AMB, Muino A, Lopez-Varela MV, Valdivia G, Lisboa C, Jardim JR, et al. A population-based cohort study on chronic obstructive pulmonary disease in Latin America: methods and preliminary results. The PLATINO Study Phase II. Arch Bronconeumol. 2014;50:10-7.
161 Menezes AM, Wehrmeister FC, Perez-Padilla R, Viana KP, Soares C, Mullerova H, et al. The PLATINO study: description of the distribution, stability, and mortality according to the Global Initiative for Chronic Obstructive Lung Disease classification from 2007 to 2017. Int J Chron Obstruct Pulmon Dis. 2017;12:1491-501.
162 Meren M, Jannus-Pruljan L, Loit HM, Polluste J, Jonsson E, Kiviloog J, et al. Asthma, chronic bronchitis and respiratory symptoms among adults in Estonia according to a postal questionnaire. Respir Med. 2001;95:954-64.
163 Miele CH, Jaganath D, Miranda JJ, Bernabe-Ortiz A, Gilman RH, Johnson CM, et al. Urbanization and Daily Exposure to Biomass Fuel Smoke Both Contribute to Chronic Bronchitis Risk in a Population with Low Prevalence of Daily Tobacco Smoking. COPD. 2016;13:186-95.
164 Milenkovic B, Mitic-Milikic M, Rebic P, Vukcevic M, Dudvarski-Ilic A, Nagorni-Obradovic L, et al. Asthma and Chronic Bronchitis Symptoms among Adult Population of Belgrade. Srpski Arhiv Za Celokupno Lekarstvo. 2011;139:149-54.
165 Minas M, Hatzoglou C, Karetsi E, Papaioannou AI, Tanou K, Tsaroucha R, et al. COPD prevalence and the differences between newly and previously diagnosed COPD patients in a spirometry program. Prim Care Respir J. 2010;19:363-70.

166 Miszkurka M, Haddad S, Langlois ÉV, Freeman EE, Kouanda S, Zunzunegui MV. Heavy burden of non-communicable diseases at early age and gender disparities in an adult population of Burkina Faso: World Health Survey. BMC Public Health. 2012;12:24.
167 Mohammadi M, Parsi B, Majd NS. Prevalence of asthma and respiratory symptoms among university students in Sari (North of Iran). Tanaffos. 2016;15:1-8.
168 Moreira GL, Gazzotti MR, Manzano BM, Nascimento O, Perez-Padilla R, Menezes AMB, et al. Incidence of chronic obstructive pulmonary disease based on three spirometric diagnostic criteria in Sao Paulo, Brazil: a nine-year follow-up since the PLATINO prevalence study. Sao Paulo Med J. 2015;133:245-51.
169 Morgan BW, Siddharthan T, Grigsby MR, Pollard SL, Kalyesubula R, Wise RA, et al. Asthma and Allergic Disorders in Uganda: A Population-Based Study Across Urban and Rural Settings. J Allergy Clin Immunol Pract. 2018;6:1580.
170 Mountjoy M, Fitch K, Boulet L-P, Bougault V, van Mechelen W, Verhagen E. Prevalence and characteristics of asthma in the aquatic disciplines. J Allergy Clin Immunol. 2015;136:588-94.
171 Mungan D, Aydin O, Mahboub B, Albader M, Tarraf H, Doble A, et al. Burden of disease associated with asthma among the adult general population of five Middle Eastern countries: Results of the SNAPSHOT program. Respir Med. 2018;139:55-64.
172 Musafiri S, van Meerbeeck J, Musango L, Brusselle G, Joos G, Seminega B, et al. Prevalence of atopy, asthma and COPD in an urban and a rural area of an African country. Respir Med. 2011;105:1596-605.
173 Nafees AA, Fatmi Z, Kadir MM, Sathiakumar N. Pattern and predictors for respiratory illnesses and symptoms and lung function among textile workers in Karachi, Pakistan. Occup Environ Med. 2013;70:99-107.
174 Nafees AA, Fatmi Z, Kadir MM, Sathiakumar N. Chronic bronchitis and chronic obstructive pulmonary disease (COPD) among textile workers in Karachi, Pakistan. J Coll Physicians Surg Pak. 2016;26:384-9.
175 Nafti S, Taright S, El Ftouh M, Yassine N, Benkheder A, Bouacha H, et al. Prevalence of asthma in North Africa: the Asthma Insights and Reality in the Maghreb (AIRMAG) study. Respir Med. 2009;103 Suppl 2:S2-11.
176 Nakao M, Yamauchi K, Ishihara Y, Omori H, Solongo B, Ichinnorov D. Prevalence and risk factors of airflow limitation in a Mongolian population in Ulaanbaatar: Cross-sectional studies. PLoS One. 2017;12:e0175557.
177 Ngui R, Lim YAL, Chow SC, de Bruyne JA, Liam CK. Prevalence of bronchial asthma among orang asli in peninsular malaysia. Med J Malaysia. 2011;66:27-31.
178 Nguyen Viet N, Yunus F, Nguyen Thi Phuong A, Dao Bich V, Damayanti T, Wiyono WH, et al. The prevalence and patient characteristics of chronic obstructive pulmonary disease in non-smokers in Vietnam and Indonesia: An observational survey. Respirology. 2015;20:602-11.
179 Nriagu J, Robins T, Gary L, Liggans G, Davila R, Supuwood K, et al. Prevalence of asthma and respiratory symptoms in south-central Durban, South Africa. Eur J Epidemiol. 1999;15:747-55.
180 Nugmanova D, Sokolova L, Feshchenko Y, Iashyna L, Gyrina O, Malynovska K, et al. The prevalence, burden and risk factors associated with bronchial asthma in commonwealth of independent states countries (Ukraine, Kazakhstan and Azerbaijan): results of the CORE study. BMC Pulm Med. 2018;18:11.

181 Nwibo AN, Ugwuja EI, Nwambeke NO, Emelumadu OF, Ogbonnaya LU. Pulmonary problems among quarry workers of stone crushing industrial site at Umuoghara, Ebonyi State, Nigeria. Int J Occup Environ Med. 2012;3:178-85.
182 Obaseki DO, Awoniyi FO, Awopeju OF, Erhabor GE. Low prevalence of asthma in sub Saharan Africa: a cross sectional community survey in a suburban Nigerian town. Respir Med. 2014;108:1581-8.
183 Obaseki DO, Erhabor GE, Gnatiuc L, Adewole OO, Buist SA, Burney PG. Chronic Airflow Obstruction in a Black African Population: Results of BOLD Study, Ile-Ife, Nigeria. COPD. 2016;13:42-9.
184 Obaseki DO, Awopeju OF, Awokola BI, Adeniyi BO, Adefuye BO, Ozoh OB, et al. Domestic solid fuel combustion in an adult population in Nigeria: A cross sectional analysis of association with respiratory symptoms, quality of life and lung function. Respir Med. 2017;130:61-8.
185 Obaseki DO, Erhabor GE, Awopeju OF, Adewole OO, Adeniyi BO, Buist EAS, et al. Reduced Forced Vital Capacity in an African Population. Prevalence and Risk Factors. Ann Am Thorac Soc. 2017;14:714-21.
186 Obel KB, Ntumba KJM, Kalambayi KP, Zalagile AP, Kinkodi KD, Munogolo KZ. Prevalence and determinants of asthma in adults in Kinshasa. PLoS ONE. 2017;12:e0176875.
187 Ornek T, Tor M, Kiran S, Atalay F. Prevalence of chronic obstructive pulmonary disease in Zonguldak province of Turkey. Tuberk Toraks. 2015;63:170-7.
188 Ory FG, Rahman FU, Katagade V, Shukla A, Burdorf A. Respiratory disorders, skin complaints, and low-back trouble among tannery workers in Kanpur, India. Am Ind Hyg Assoc J. 1997;58:740-6.
189 Ozdemir N, Ucgun I, Metintas S, Kolsuz M, Metintas M. The prevalence of asthma and allergy among university freshmen in Eskisehir, Turkey. Respir Med. 2000;94:536-41.
190 Padhi BK, Padhy PK. Assessment of Intra-urban Variability in Outdoor Air Quality and its Health Risks. Inhal Toxicol. 2008;20:973-9.
191 Pan J, Xu L, Lam TH, Jiang CQ, Zhang WS, Jin YL, et al. Association of adiposity with pulmonary function in older Chinese: Guangzhou Biobank Cohort Study. Respir Med. 2017;132:102-8.
192 Papageorgiou N, Gaga M, Marossis C, Reppas C, Avarlis P, Kyriakou M, et al. Prevalence of asthma and asthma-like symptoms in Athens, Greece. Respir Med. 1997;91:83-8.
193 Parasuramalu BG, Huliraj N, Rudraprasad BM, Prashanth Kumar SP, Gangaboraiah, Ramesh Masthi NR. Prevalence of bronchial asthma and its association with smoking habits among adult population in rural area. Indian J Public Health. 2010;54:165-8.
194 Parasuramalu BG, Huliraj N, Prashanth Kumar SP, Gangaboraiah, Ramesh Masthi NR, Srinivasa Babu CR. Prevalence of chronic obstructive pulmonary disease and its association with tobacco smoking and environmental tobacco smoke exposure among rural population. Indian J Public Health. 2014;58:45-9.
195 Pefura-Yone EW, Kengne AP, Balkissou AD, Boulleys-Nana JR, Efe-de-Melingui NR, Ndjeutcheu-Moualeu PI, et al. Prevalence of asthma and allergic rhinitis among adults in Yaounde, Cameroon. PloS one. 2015;10:e0123099.
196 Perez-Padilla R, Fernandez R, Varela MVL, de Oca MM, Muino A, Talamo C, et al. Airflow Obstruction in Never Smokers in Five Latin American Cities: The PLATINO Study. Arch Med Res. 2012;43:159-65.

197 Pothirat C, Chaiwong W, Phetsuk N, Pisalthanapuna S, Chetsadaphan N, Inchai J. A comparative study of COPD burden between urban vs rural communities in northern Thailand. Int J Chron Obstruct Pulmon Dis. 2015;10:1035-42.

198 Pothirat C, Phetsuk N, Liwsrisakun C, Bumroongkit C, Deesomchok A, Theerakittikul T. Major Chronic Respiratory Diseases in Chiang Mai: Prevalence, Clinical Characteristics, and Their Correlations. J Med Assoc Thai. 2016;99:1005-13.

199 Priftanji AV, Qirko E, Layzell JCM, Burr ML, Fifield R. Asthma and allergy in Albania. Allergy. 1999;54:1042-7.

200 Quansah R, Bend JR, Abdul-Rahaman A, Armah FA, Luginaah I, Essumang DK, et al. Associations between pesticide use and respiratory symptoms: A cross-sectional study in Southern Ghana. Environmental Research. 2016;150:245-54.

201 Queiroz MCdCAMd, Moreira MAC, Rabahi MF. Subdiagnóstico de DPOC na atenção primária em Aparecida de Goiânia, Goiás. Brazillian Journal of Pulmonology. 2012;38:692-9.

202 Rabahi MF, Pereira SA, Silva JLR, de Rezende AP, da Costa AC, Correa KD, et al. Prevalence of chronic obstructive pulmonary disease among patients with systemic arterial hypertension without respiratory symptoms. Int J Chron Obstruct Pulmon Dis. 2015;10:1525-9.

203 Radwan GN, Latif S, Amin N, Galal D, Aziz M, Attia E. Occupational exposure to second hand smoke and respiratory and sensory symptoms: a cross-sectional survey of hospital workers in Egypt. International Journal of Occupational Medicine and Environmental Health. 2014;27:60-70.

204 Rahhal BM, Abu Rmileh J, Kalouti N, Murrar S. Assessment of Respiratory Health Indicators among Agricultural Workers Exposed to Pesticides: A Cross Sectional Study from Palestine. Mor J Chem. 2017;5:266-71.

205 Rahimi Rad MH, Rezaei MK, Abdollahi N, Hatami K. Smoking and asthma symptoms among University students. Tanaffos. 2007;6:53-8.

206 Rahimi-Rad MH, Gaderi-Pakdel F, Salari-Lak S. Smoking and asthma in 20-44-year-old adults in Urmia, Islamic Republic of Iran. East Mediterr Health J. 2008;14:6-16.

207 Rahman A, Saeed A, Ali M. Prevalence of chronic obstructive pulmonary disease as occupational lung disease among brick kiln workers. Pakistan Journal of Medical and Health Sciences. 2013;7:618-21.

208 Ramirez-Venegas A, Velazquez-Uncal M, Perez-Hernandez R, Guzman-Bouilloud NE, Falfan-Valencia R, Mayar-Maya ME, et al. Prevalence of COPD and respiratory symptoms associated with biomass smoke exposure in a suburban area. Int J Chron Obstruct Pulmon Dis. 2018;13:1727-34.

209 Rao S, Ashok NC, Jain T, Anuradha R, Dhar M. Influence of associated factors in the prevalence of asthma: A community based study in Mysore. J Clin Diagn Res. 2011;5:721-4.

210 Regalado J, Perez-Padilia R, Sansores R, Ramirez JIP, Brauer M, Pare P, et al. The effect of biomass burning on respiratory symptoms and lung function in rural Mexican women. Am J Respir Crit Care Med. 2006;174:901-5.

211 Rose D, Mannino DM, Leaderer BP. Asthma prevalence among US adults, 1998-2000: Role of Puerto Rican ethnicity and behavioral and geographic factors. American Journal of Public Health. 2006;96:880-8.

212 Rumana HS, Sharma RC, Beniwal V, Sharma AK. A retrospective approach to assess human health risks associated with growing air pollution in urbanized area of Thar Desert, western Rajasthan, India. J Environ Health Sci Eng. 2014;12:23.

213 Sakar A, Yorgancioglu A, Dinc G, Yuksel H, Celik P, Dagyildizi L, et al. The prevalence of asthma and allergic symptoms in Manisa, Turkey (A western city from a country bridging Asia and Europe). Asian Pac J Allergy Immunol. 2006;24:17-25.

214 Sallaoui R, Chamari K, Chtara M, Alaranta A, Manai Y, Ghedira H, et al. Asthma in Tunisian elite athletes. Int J Sports Med. 2007;28:571-5.

215 Sansores RH, Ramirez-Venegas A, Hernandez-Zenteno R, Mayar-Maya ME, Perez-Bautista OG, Velazquez Uncal M. Prevalence and diagnosis of chronic obstructive pulmonary disease among smokers at risk. A comparative study of case-finding vs. screening strategies. Respir Med. 2013;107:580-6.
216 Santos SR, Lizzi ES, Vianna EO. Characteristics of undiagnosed COPD in a senior community center. Int J Chron Obstruct Pulmon Dis. 2014;9:1155-61.
217 Saraclar Y, Cetinkaya F, Tuncer A, Kalayci O, Adalioglu G, Sekerel BE, et al. The prevalence of self-reported asthma and respiratory symptoms in Ankara, Turkey. Respir Med. 1997;91:461-3.
218 Schiavi E, Stirbulov R, Vecino RH, Mercurio S, Di Boscio V, Equipo P. COPD Screening in Primary Care in Four Latin American Countries: Methodology of the PUMA Study. Arch Bronconeumol. 2014;50:469-74.
219 Sembajwe G, Cifuentes M, Tak SW, Kriebel D, Gore R, Punnett L. National income, self-reported wheezing and asthma diagnosis from the World Health Survey. Eur Respir J. 2010;35:279-86.
220 Shahzad K, Akhtar S, Mahmud S. Prevalence and determinants of asthma in adult male leather tannery workers in Karachi, Pakistan: a cross sectional study. BMC Public Health. 2006;6:292.
221 Shaikh S, Nafees AA, Khetpal V, Jamali AA, Arain AM, Yousuf A. Respiratory symptoms and illnesses among brick kiln workers: a cross sectional study from rural districts of Pakistan. BMC Public Health. 2012;12:1-6.
222 Sharifi H, Masjedi MR, Emami H, Ghanei M, Eslaminejad A, Radmand G, et al. Burden of obstructive lung disease study in Tehran: Prevalence and risk factors of chronic obstructive pulmonary disease. Lung India. 2015;32:572-7.
223 Shishani K. Respiratory symptoms reported by adults living in an air polluted area in Jordan. Jordan Med J. 2006;40:21-8.
224 Sichletidis L, Tsiotsios I, Gavriilidis A, Chloros D, Kottakis I, Daskalopoulou E, et al. Prevalence of chronic obstructive pulmonary disease and rhinitis in northern Greece. Respiration. 2005;72:270-7.
225 Sichletidis L, Spyratos D, Papaioannou M, Chloros D, Tsiotsios A, Tsagaraki V, et al. A combination of the IPAG questionnaire and PiKo-6 flow meter is a valuable screening tool for COPD in the primary care setting. Prim Care Respir J. 2011;20:184-9.
226 Siddanagoudra SP. Respiratory morbidity in spray paint workers in an automobile sector. Indian J Public Health Res Dev. 2014;5:10-5.
227 Siddharthan T, Grigsby MR, Goodman D, Chowdhury M, Rubinstein A, Irazola V, et al. Association between Household Air Pollution Exposure and Chronic Obstructive Pulmonary Disease Outcomes in 13 Low- and Middle-Income Country Settings. Am J Respir Crit Care Med. 2018;197:611-20.
228 Sigari N, Rahimi E, Yazdanpanah K, Sharifian A. Prevalence of asthma and rhinitis in bakery workers in the city of Sanandaj, Iran. Iran J Allergy Asthma Immunol. 2007;6:215-8.
229 Singh T, Bello B, Jeebhay MF. Risk factors associated with asthma phenotypes in dental healthcare workers. Am J Ind Med. 2013;56:90-9.
230 Sinha B, Vibha, Singla R, Chowdhury R. An epidemiological profile of chronic obstructive pulmonary disease: A community-based study in Delhi. J Postgrad Med. 2017;63:29-35.
231 Smeeton NC, Rona RJ, Oyarzun M, Diaz PV. Agreement between responses to a standardized asthma questionnaire and a questionnaire following a demonstration of asthma symptoms in adults. Am J Epidemiol. 2006;163:384-91.
232 Smith M, Li L, Augustyn M, Kurmi O, Chen J, Collins R, et al. Prevalence and correlates of airflow obstruction in 317,000 never-smokers in China. Eur Respir J. 2014;44:66-77.

233 Sobrino E, Irazola VE, Gutierrez L, Chen C-S, Lanas F, Calandrelli M, et al. Estimating prevalence of chronic obstructive pulmonary disease in the Southern Cone of Latin America: how different spirometric criteria may affect disease burden and health policies. BMC Pulm Med. 2017;17:187.
234 Sonia T, Meriem M, Yacine O, Nozha BS, Nadia M, Bechir L, et al. Prevalence of asthma and rhinitis in a Tunisian population. Clin Respir J. 2018;12:608-15.
235 Sonomjamts M, Dashdemberel S, Logii N, Nakae K, Chigusa Y, Ohhira S, et al. Prevalence of asthma and allergic rhinitis among adult population in Ulaanbaatar, Mongolia. Asia Pac Allergy. 2014;4:25-31.
236 Soongkhang I, Laohasiriwong W. Respiratory Tract Problems among Wood Furniture Manufacturing Factory Workers in the Northeast of Thailand. Kathmandu Univ Med J (KUMJ). 2015;13:125-9.
237 Stankovic A, Nikolic M, Arandjelovic M. Effects of indoor air pollution on respiratory symptoms of non-smoking women in Nis, Serbia. Multidiscip Respir Med. 2011;6:351-5.
238 Stephen P, Mahalakshmy T, Manju R, Laksham KB, Subramani S, Panda K, et al. High Prevalence of Chronic Respiratory Symptoms among Autorickshaw Drivers of Urban Puducherry, South India. Indian J Occup Environ Med. 2018;22:40-4.
239 Sy DQ, Thanh Binh MH, Quoc NT, Hung NV, Quynh Nhu DT, Bao NQ, et al. Prevalence of asthma and asthma-like symptoms in Dalat Highlands, Vietnam. Singapore Med J. 2007;48:294-303.
240 Tageldin MA, Nafti S, Khan JA, Nejjari C, Beji M, Mahboub B, et al. Distribution of COPD-related symptoms in the Middle East and North Africa: Results of the BREATHE study. Respir Med. 2012;106:S25-S32.
241 Tarraf H, Aydin O, Mungan D, Albader M, Mahboub B, Doble A, et al. Prevalence of asthma among the adult general population of five Middle Eastern countries: results of the SNAPSHOT program. BMC Pulm Med. 2018;18:14.
242 Tiwari RR, Zodpey SP, Deshpande SG, Ughade SN, Vasudeo ND. Respiratory morbidity in handloom weavers. Indian J Occup Environ Med. 1999;3:71-3.
243 To T, Stanojevic S, Moores G, Gershon AS, Bateman ED, Cruz AA, et al. Global asthma prevalence in adults: findings from the cross-sectional world health survey. BMC Public Health. 2012;12:204.
244 Toru U, Arbak PM, Suner KO, Yavuz O, Karatas N. Relationship between Respiratory Tract Complaints, Functional Status, and Smoking in Hairdressers, Auto Painters, and Carpenters. The Scientific World Journal. 2014.
245 Townend J, Minelli C, Mortimer K, Obaseki DO, Al Ghobain M, Cherkaski H, et al. The association between chronic airflow obstruction and poverty in 12 sites of the multinational BOLD study. Eur Respir J. 2017;49:1601880.
246 Tug T, Acik Y. Prevalence of asthma, asthma-like and allergic symptoms in the urban and rural adult population in Eastern Turkey. Asian Pac J Allergy Immunol. 2002;20:209-15.
247 Tu T, Acik Y, Tu E. Prevalence of asthma and asthma-like and allergic symptoms in the urban adult population of Elazig. Turk J Med Sci. 2002;32:243-9.
248 Tzanakis N, Anagnostopoulou U, Filaditaki V, Christaki P, Siafakas N, Society CgotHT. Prevalence of COPD in Greece. Chest. 2004;125:892-900.
249 Ugurlu E, Oncel SB, Evyapan F. Symptom prevalence and risk factors for asthma at the rural regions of Denizli, Turkey. J Thorac Dis. 2014;6:452-8.

250 Uthaisangsook S. Risk factors for development of asthma in Thai adults in Phitsanulok: a university-based study. Asian Pac J Allergy Immunol. 2010;28:23-8.
251 Uthaisangsook S. Prevalence of asthma, rhinitis, and eczema in the university population of Phitsanulok, Thailand. Asian Pac J Allergy Immunol. 2007;25:127-32.
252 Valenti C, Pozzi P, Busia A, Mazza R, Bossi P, De Marco C, et al. Respiratory illness and air pollution from the steel industry: the case of Piquia de Baixo, Brazil (Preliminary report). Multidiscip Respir Med. 2016;11:1-7.
253 van Gemert F, Kirenga B, Chavannes N, Kamya M, Luzige S, Musinguzi P, et al. Prevalence of chronic obstructive pulmonary disease and associated risk factors in Uganda (FRESH AIR Uganda): a prospective cross-sectional observational study. Lancet Glob Health. 2015;3:e44-51.
254 Varona LL, Alava HDA, Abong JM, Castor MAR, De Leon JC, Kwong SL. Prevalence of asthma among filipino adults based on the national nutrition and health survey (NNHeS). Philipp J Intern Med. 2014;52:1-7.
255 Vichyanond P, Sunthornchart S, Singhirannusorn V, Ruangrat S, Kaewsomboon S, Visitsunthorn N. Prevalence of asthma, allergic rhinitis and eczema among university students in Bangkok. Respir Med. 2002;96:34-8.
256 Viinanen A, Munhbayarlah S, Zevgee T, Narantsetseg L, Naidansuren T, Koskenvuo M, et al. Prevalence of asthma, allergic rhinoconjunctivitis and allergic sensitization in Mongolia. Allergy. 2005;60:1370-7.
257 Viswanathan K, Rakesh PS, Balakrishnan S, Shanavas A, Dharman V. Prevalence of chronic respiratory diseases from a rural area in Kerala, southern India. Indian J Tuberc. 2018;65:48-51.
258 Waked M, Khayat G, Salameh P. Chronic obstructive pulmonary disease prevalence in Lebanon: A cross-sectional descriptive study. Clinical Epidemiology. 2011;3:315-23.
259 Waked M, Salame J, Khayat G, Salameh P. Correlates of COPD and chronic bronchitis in nonsmokers: Data from a cross-sectional study. Int J Chron Obstruct Pulmon Dis. 2012;7:577-85.
260 Walraven GEL, Nyan OA, Van der Sande MAB, Banya WAS, Ceesay SM, Milligan PJM, et al. Asthma, smoking and chronic cough in rural and urban adult communities in The Gambia. Clin Exp Allergy. 2001;31:1679-85.
261 Wang XR, Zhang HX, Sun BX, Dai HL, Hang JQ, Eisen EA, et al. A 20-year follow-up study on chronic respiratory effects of exposure to cotton dust. Eur Respir J. 2005;26:881-6.
262 Wang DX, Xiao W, Ma DD, Zhang YK, Wang Q, Wang C, et al. Cross-sectional epidemiological survey of asthma in Jinan, China. Respirology. 2013;18:313-22.
263 Wang J, Li S, Wang S, Shang K. Effects of Long-Term Dust Exposure on Human Respiratory System Health in Minqin County, China. Archives of Environmental and Occupational Health 2015;70:225-31.
264 Wang C, Xu J, Yang L, Xu Y, Zhang X, Bai C, et al. Prevalence and risk factors of chronic obstructive pulmonary disease in China (the China Pulmonary Health [CPH] study): a national cross-sectional study. Lancet. 2018;391:1706-17.
265 Wilson D, Takahashi K, Pan GW, Chan CC, Zhang SJ, Feng YP, et al. Respiratory symptoms among residents of a heavy-industry province in China: Prevalence and risk factors. Respir Med. 2008;102:1536-44.

266 Xiao GB, Morinaga K, Wang RY, Xu LR, Ma ZH, Zhang X, et al. Lung disorders of workers exposed to rush smear dust in China. Ind Health. 2006;44:556-63.

267 Xu X, Niu T, Chen C, Wang B, Jin Y, Yang J, et al. Association of airway responsiveness with asthma and persistent wheeze in a Chinese population. Chest. 2001;119:691-700.

268 Xu F, Yin X, Zhang M, Shen H, Lu L, Xu Y. Prevalence of physician-diagnosed COPD and its association with smoking among urban and rural residents in regional mainland China. Chest. 2005;128:2818-23.

269 Yamamoto M, Tsurugi Y, Matsuda S, Luong NA. Occupational exposure, environmental pollution and chronic respiratory symptoms in Vietnam. Journal of UOEH. 2000;22:325-37.

270 Yemaneberhan H, Bekele Z, Venn A, Lewis S, Parry E, Britton J. Prevalence of wheeze and asthma and relation to atopy in urban and rural Ethiopia. Lancet. 1997;350:85-90.

271 Yildiz T, Topcu F, Celik L, Akyildiz L, Ates G, Durak D. Effect of passive smoking on the development of chronic obstructive pulmonary disease in southeastern Turkey. Turk J Med Sci. 2010;40:349-55.

272 Yin P, Zhang M, Li Y, Jiang Y, Zhao W. Prevalence of COPD and its association with socioeconomic status in China: findings from China Chronic Disease Risk Factor Surveillance 2007. BMC Public Health. 2011;11:586.

273 Yingratanasuk T, Seixas N, Barnhart S, Brodkin D. Respiratory health and silica exposure of stone carvers in Thailand. Int J Occup Environ Health. 2002;8:301-8.

274 Zhang LX, Enarson DA, He GX, Li B, Chan-Yeung M. Occupational and environmental risk factors for respiratory symptoms in rural Beijing, China. Eur Respir J. 2002;20:1525-31.

275 Zhang W, Chen X, Ma L, Wu J, Zhao L, Kuang H, et al. Epidemiology of bronchial asthma and asthma control assessment in Henan Province, China. Transl Respir Med. 2014;2:1-7.

276 Zhang FY, Hang JQ, Zheng BY, Su L, Christiani DC. The changing epidemiology of asthma in Shanghai, China. J Asthma. 2015;52:465-70.

277 Abraham E. Prevalence of chronic obstructive pulmonary disease in China (vol 176, pg 753, 2007). Am J Respir Crit Care Med. 2007;176:1169-.

278 Zhou Y, Yao W, Chen P, Kang J, Huang S, Chen B, et al. COPD in Chinese nonsmokers. Eur Respir J. 2009;33:509-18.

279 Zoller T, Mfinanga EH, Zumba TB, Asilia PJ, Mutabazi EM, Wimmersberger D, et al. Chronic airflow obstruction in Tanzania - a cross-sectional study. BMC Pulm Med. 2018;18:11.

280 Zubair T, Abbassi A, Khan OA. Early Detection of Chronic Obstructive Pulmonary Disease in Apparently Healthy Attendants of Tertiary Care Hospital and Assessment of its Severity. Journal of the College of Physicians and Surgeons--Pakistan : JCPSP. 2017;27:296-300.

281 Zuskin E, Smolej Narancic N, Skaric-Juric T, Barbalic M, Rudan P, Kujundzic-Tiljak M, et al. Chronic respiratory symptoms in Croatian Adriatic island metapopulations. Croat Med J. 2006;47:627-34.
